# Quantitative sustainability assessment of household food waste management in the Amsterdam Metropolitan Area

**DOI:** 10.1016/j.resconrec.2020.104854

**Published:** 2020-09

**Authors:** Davide Tonini, Alexander Wandl, Kozmo Meister, Pablo Muñoz Unceta, Sue Ellen Taelman, David Sanjuan-Delmás, Jo Dewulf, Dries Huygens

**Affiliations:** a European Commission, Joint Research Centre (JRC), Seville, Spain; b Technical University of Delft, Delft, the Netherlands; c Faculty of Bioscience Engineering, Department of Green Chemistry and Technology, Ghent University, Ghent, Belgium

**Keywords:** Stakeholder, Waste collection, LCA, Local impacts, Multi-criteria decision analysis, Circular Economy

## Abstract

Food waste represents the largest fraction of the municipal solid waste generated in Europe and its management is associated to suboptimal performance in environmental, health, and social dimensions. By processing detailed multi-fold local data as part of a comprehensive and broadly understandable sustainability framework, this study quantifies the environmental and socio-economic impacts of household food waste management in the Amsterdam Metropolitan Area based on priorities set by local stakeholders. Five alternative short-term management options have been assessed against the current system, relying on poor separate collection and incineration. Four options involve separate collection of food waste followed by biological treatments (home/centralised composting and anaerobic digestion) while one involves a mix of separate collection and centralised mechanical-biological treatment followed by anaerobic digestion. Among these, separate collection followed by anaerobic digestion coupled with effective nutrient and energy recovery is, according to our findings, the preferred option to improve the sustainability of the current system in all dimensions considered, except for the economic pillar due to the collection costs. Home and centralised composting as well as mechanical-biological treatment are associated to more adverse impacts based on our findings. The study informs local stakeholders and authorities on the potential consequences of their options, thereby allowing them to make sound choices for a future waste and circular economy strategy.

## Highlights

• Multi-fold local & global data collected and processed into a comprehensive framework
• Anaerobic digestion with effective nutrient recovery appears the best option
• All alternative strategies to incineration increase food waste management costs
• Aggregation makes findings accessible to the widest possible audience and stakeholder
• The study informs stakeholders and authorities on the consequences of their options

AMA: Amsterdam Metropolitan Area;
AoP: Area of Protection;
cAD: scenario based on centralised anaerobic digestion;
cAD-PP: scenario based on centralised anaerobic digestion followed by advanced post-processing of digestate;
CAPEX: capital expenditures;
cCP: scenario based on centralised composting;
EH: ecosystem health;
FU: Functional Unit;
hCP: scenario based on home composting;
HH: human health;
HW: human well-being;
LCA: life cycle assessment;
MBT: scenario based on mechanical biological treatment;
MCDA: multi-criteria decision analysis;
MSW: municipal solid waste;
NSC-FW: non-separately collected food waste;
OELEX: end-of-life expenditures;
OPEX: operational expenditures;
NR: natural resource;
PR: prosperity
REF: reference scenario (*status quo*);
SC-FW: separately collected food waste;
SME: small and medium enterprises;
WMS: waste management system.

## 1 Introduction

Food waste is the largest material fraction of the municipal solid waste (MSW) generated in Europe (with a share of 30-50%; Treadwell, et al., 2018). The improper or suboptimal management of food waste causes environmental, health and social impacts (Manfredi and Christensen, 2009; Manfredi et al., 2010) or lost opportunities for increasing environmental and socio-economic returns (amongst others Eriksson et al., 2016; Schanes et al., 2018; Albizzati et al., 2019). The European Commission prioritises prevention measures to meet the Sustainable Development Goal #12 (halving food waste per capita by 2030 and reducing food losses in the production/supply sectors; United Nations, 2015), but likewise promotes the separate collection of the generated food waste and the recovery of resources (European Parliament and the Council, 2018; European Commission, 2015). In the latest years, the EU is moving away from landfilling as a MSW treatment method, resulting in increases in the share of MSW that is recycled and incinerated (Eurostat, 2019). However, the 60-65% policy target set out for the years 2030-2035 on the amount of MSW prepared and sent for reuse or recycling is only likely to be met when incineration of food waste is avoided. While a general management hierarchy is proposed (European Parliament and the Council, 2018), the choice of the management scheme can be situation-dependent to ensure the environmental, economic, and social sustainability at the local level. To this purpose, application of life cycle thinking is recommended (European Parliament and the Council, 2008).

Many studies have tackled the performance of municipal food waste management schemes through life cycle assessment (most recently: Thyberg and Tonjes, 2017; Oldfield, White and Holden, 2018; Slorach et al., 2019; Yeo et al., 2019) and costing (among the others: Kim et al., 2011; Martinez-Sanchez et al., 2016; Slorach et al., 2019). However, few studies performed a holistic sustainability assessment encompassing environmental, economic and social pillars using primary, site-specific data and involving local stakeholders in the definition of the sustainability framework as recommended by best practices (UNEP, 2011; Taelman et al., 2018). Despite the numerous frameworks proposed in the literature, data collection challenges and possible other factors have limited the number of analytical studies (to the best of our knowledge: Gabbay de Souza et al., 2016, Zijp et al., 2017, Millward-Hopkins et al., 2018, Di Maria and Sisani, 2019, Stone et al. 2019, and Zhou et al., 2019). Moreover, for the specific case of household and/or municipal food waste, no such studies are available. Reviewing the literature, we identified four main issues that can further enhance the full implementation of sustainability frameworks. This first issue is a systematic stakeholder involvement in identifying the relevant impact categories to be included in the framework. We found only a few studies that clearly documented a systematic involvement of the stakeholders in singling out the relevant impact categories for the case under assessment e.g. through dedicated questionnaires or workshops, i.e. Gabbay de Souza et al. 2016 and Zijp et al. 2017. In most of the cases, the impact categories were ultimately selected by the authors considering the perception of the affected parties but without their direct and systematic involvement based on their priorisation of effects (e.g. Stone et al. 2019, Millward-Hopkins et al., 2018 and Zhou et al., 2019). Secondly, an appropriate inventory data collection may enhance the reliability of the findings. While many frameworks have been proposed at a theoretical level, few studies documented and reported a comprehensive data collection to effectively apply the proposed framework (e.g. Zhou et al. 2019). This is especially relevant when local impacts are addressed, e.g. disamenities, space consumption or employment. Due to limited data availability and related challenges, most studies relied on existing life cycle inventory datasets corrected with their own assumptions (Gabbay de Souza et al., 2016 and Di Maria and Sisani, 2019). Others rather focused on method development with less enphasis on data collection being exploratory/preliminary studies (Zijp et al., 2017 or Millward-Hopkins et al., 2018). Thirdly, a robust treatment of the uncertainty is often omitted or only partially considered by performing selected scenario analyses (i.e. varying key scenario assumptions, one-at-the-time). In this respect, only a few studies treated uncertainty, e.g. through parameter propagation (Di Maria and Sisani, 2019) or scenario analyses (Stone et al., 2019; Zhou et al., 2018). Lastly, a final aggregation of the results to facilitate the synthesis and communication is performed only in a limited number of studies, notably Stone et al. (2019), Di Maria and Sisani (2019), and Gabbay de Souza et al*.* 2016.

Bearing in mind these issues, we build further upon previous works and strive to advance the current state-of-the-art knowledge in the field of sustainability assessment by: i) collecting detailed local data to quantify the environmental, economic, and social indicators outlined in Taelman et al., 2019 based on priorities of local stakeholders; ii) evaluating alternative food waste management options and recommending sustainable solutions to support local strategies and policies, and iii) strengthening the results with uncertainty analyses including parameter propagation and scenario analyses applying recent developments in the methodology (Bisinella et al., 2016) and to the extent possible given data limitations. To this end we quantify the sustainability of the *status quo* and five alternative household food waste management scenarios for the case of the Amsterdam Metropolitan Area (AMA).

## 2 Methods

### 2.1 Focus Area

**Figure 1:**
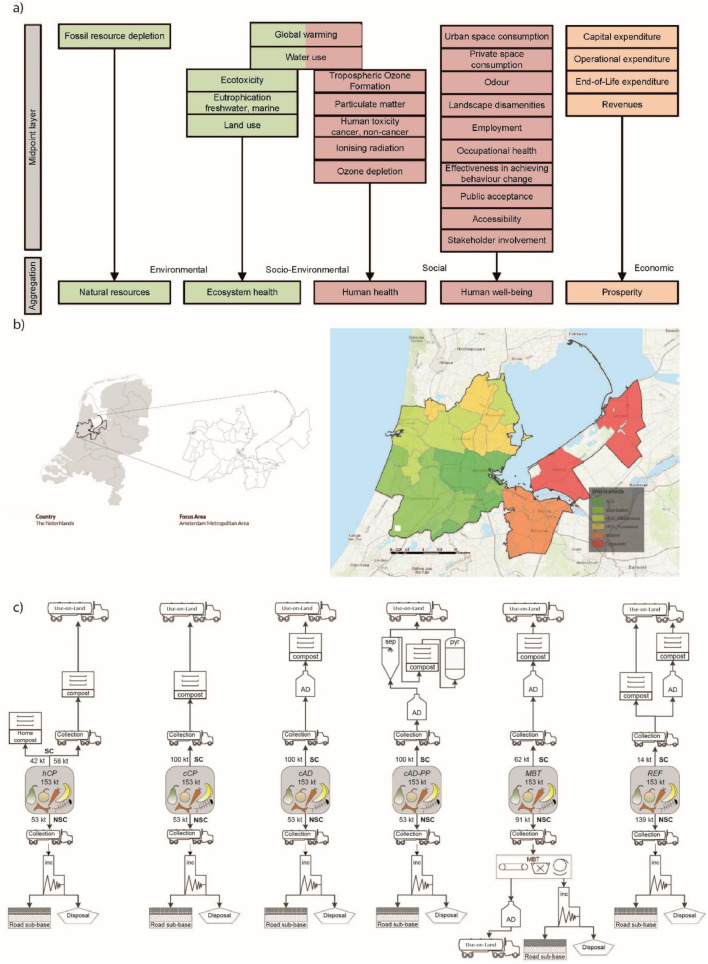
Illustration of: a) the sustainability framework as applied in the study (27 indicators covering twenty-five impact categories; cfr. Taelman et al., 2019), b) the Amsterdam Metropolitan Area as focus area of the assessment, and c) the *status quo* and five alternative scenarios assessed for the management of the food waste annually generated in the focus area by households and SMEs. The scenarios are named (left-to-right): I) *hCP* (home composting), II) *cCP*: (centralised composting); III) c*AD*: centralised anaerobic digestion; IV) *cAD-PP*: centralised anaerobic digestion with post-processing of the digestate; V) *MBT*: mechanical-biological treatment; VI) *REF*: reference scenario (*status quo*). Also, NSC-FW: non-separately collected food waste; pyr: pyrolysis; SC-FW: separately collected food waste; sep: separation.

Our focus area is the AMA (Figure 1b), a collaboration of two provinces (Noord-Holland and Flevoland), 32 municipalities and the Amsterdam Transport Region. It covers the area around the city of Amsterdam and forms the north wing of the Randstad. With 2.4 million inhabitants, two airports, a large seaport, the financial centre of the Netherlands and the flower auction of Aalsmeer it is one of the top five economic regions in Europe. One of the key ambitions formulated in the development program of the AMA is to play a pioneering role in the knowledge and circular economy (Metropoolregio Amsterdam, 2019). Currently, the management of food waste generated by households is characterised by low separate collection and mostly relies on incineration with energy recovery (Figure 1c). To promote circular economy and fulfil related recycling targets, the city authorities have, among other initiatives, committed to improve the separate collection of food waste and reduce incineration by promoting alternatives such as anaerobic digestion and composting to recover nutrients and carbon in a more closed urban-rural system (LAP, 2010).

### 2.2 Scope and functional unit

The functional unit (FU) is the management of food waste annually generated by the households and small-and-medium-enterprises (SMEs) in the AMA, totalling 153,310 t of wet weight. The assessment is performed applying the framework developed in Taelman et al., 2019 encompassing five areas-of-protection (AoPs) at the endpoint level, with a total of 27 indicators for 25 different impact categories at midpoint level, either environmental, social or economically oriented (Figure 1a). This involved a two-stage participatory process whereby local and European stakeholders were engaged to: i) identify the relevant sustainability impact categories through questionnaires (details in Taelman et al., 2019) and ii) local stakeholders proposed, as part of a two year long co-creative living lab, a set of waste management scenarios to be assessed (Remoy et al., 2018). The stakeholder groups were government, research and education, non-governmental organisations, private sector, waste management, and non-waste infrastructure operators. A subsequent scientific procedure was followed to recommend specific state-of-the-art indicators for each impact category alongside their detailed calculation method (Table C.16; details in Taelman et al., 2019). Additionally, the framework proposes a final aggregation of the results into a ranking of the scenarios from best to worst at the level of the AoPs. The ranking is achieved by applying multi-criteria decision analysis (MCDA) based on the implementation of the ELECTRE II method (Figueira et al., 2005, 2010; Lima and Salazar Soares, 2011). For this, a dedicated excel-model was developed and is available as supporting information in Taelman et al., 2019. For a broader perspective on the options available for aggregation and on the methodological details of the method applied in this study, the reader is referred to Taelman et al., 2019 and Tonini et al., (2018b). The assessment applies a consequential approach (Weidema, 2003; Ekvall and Weidema, 2004; Weidema et al., 2009) striving to address the consequences incurred by the future changes in the waste management system compared to the *status quo*. The methodology to quantify annualised unit-costs (as capital, operational, and End-of-Life expenditures; CAPEX, OPEX, and OELEX) follows the approach of Martinez-Sanchez et al. (2015). The methodology to calculate the social indicators Public Acceptance, Stakeholder Involvement, Accessibility to waste management system (WMS) and Landscape Disamenities is detailed in Appendix B (see also Taelman et al., 2019). Accidents were quantified by multiplying the overall labour required for each scenario by the appropriate accident rate. Notice that emissions are accounted considering a time horizon of 100y after disposal. In Global Warming, the uptake/release of CO_2_ biogenic was assigned a characterization factor equal to 0, while the eventually non-emitted CO_2_ biogenic was assigned a factor equal to -1, following common practice for short-live biomass. The assessment was facilitated with the tool EASETECH (Clavreul et al., 2014).

### 2.3 Scenarios assessed

We assessed the reference scenario (*status quo*) and five alternative scenarios focused on increased food waste separation and selected after stakeholder consultation (see Remoy *et al.*, 2018; Figure 1c). While the reference (*REF*) largely relies on poor food waste separation and subsequent incineration with energy recovery, scenarios I-to-V differ from *REF* either by having an improved separate collection system for food waste and/or alternative treatment pathways to incineration. More specifically, the following scenarios were considered: I) *hCP*: separate collection followed by home composting of the food waste generated by the households having a private garden and separate collection followed by centralised composting for the food waste generated by the remaining households (not having private garden) alongside SMEs (the sum of these two flows makes the separately collected food waste; SC-FW); for both households types, the food waste capture rate was assumed to be 65% to ensure fulfilling the EU recycling target of 55% for 2025 considering inefficiencies/rejects (European Parliament and the Council, 2018); the non-separately collected food waste (NSC-FW) was assumed to be collected with the mixed waste and sent to incineration following current practice. II) *cCP*: separate collection of the food waste generated followed by centralised composting; the assumptions on capture rate and treatment of NSC-FW are the same as for *hCP*. III) *cAD*: separate collection of the food waste generated followed by centralised anaerobic digestion and post-composting; the assumptions on capture rate and treatment of NSC-FW are the same as for *hCP*. IV) *cAD-PP*: as scenario *cAD*, but the digestion is followed by advanced post-treatments aiming to produce concentrated fertilising and amending products, in the form of ammonium sulphate and biochar, respectively. V) *MBT*: as scenario *cAD* with the difference that no separate collection is performed in the city centre (wasteshed namely AEB; Figure 1b); instead, the mixed waste collected in this area and elsewhere is sent to an advanced mechanical-biological treatment (MBT) to recover the biomass fraction in the form of a bioliquid that undergoes anaerobic digestion (Tonini et al., 2013). VI) *REF*: representing the *status quo* where most of the food waste is incinerated together with the mixed waste.

### 2.4 System boundary

For all scenarios assessed, the system boundary includes all the activities involved in the life cycle of the generated waste: collection, treatment, transportation of waste, treatment residues and/or intermediate products to end-use (e.g. ashes, digestate, compost), or eventual final disposal (e.g. backfilling), in line with Figure 1b. Activities (e.g. effort and time spent by households) and goods (e.g. garbage bins and bags) associated with in-house source segregation of the waste have been disregarded. Following common practice in LCA of waste systems, the secondary products and services generated alongside the management of the waste (i.e. the FU) were credited by assuming substitution of corresponding market products or services. These products/services were identified in the market as marginal products/services for the area under assessment, i.e. those capable to respond to changes in demand (Weidema et al., 2009); an example of system boundary is illustrated in Figure B.1 (Appendix B). On this basis, electricity provision was assumed as the Dutch marginal mix for the period 2015-2030 (24% biomass assumed as wood pellets, 53% wind energy, 13% natural gas, 10% solar) as reported in Ecoinvent centre, 2019 on the basis of European Commission (2016); likewise, a marginal heat mix based on natural gas and heat pumps (with shares equalling 46% and 54%, respectively) was elaborated on the basis of the information provided in Heat Roadmap Europe for the business-as-usual future heat supply mix of the Netherlands (Nijs et al., 2016). With respect to the production of gaseous fuel, such as upgraded biogas (i.e. with natural gas-quality and injected into the gas grid), we assumed a 1-to-1 substitution of natural gas extraction, (long-distance) distribution, and combustion based on the energy content. With respect to NPK mineral fertilisers, we relied on the choices justified in previous studies assuming an average EU mix (urea 24.5%, ammonium nitrate 27%, calcium ammonium nitrate 33%, and urea-ammonium nitrate 15.5%), diammonium phosphate, and potassium chloride as marginal N, P, and K mineral fertilisers (Tonini et al., 2016; Tonini et al., 2019). In line with Dutch legislation, the application rates for the fertilising materials/products derived from the food waste were applied on land at rates that do not exceed plant nutrient demands. Therefore, it was assumed that bioavailable NPK in those secondary materials/products substituted virgin mineral NPK fertilisers in a 1-to-1 ratio. Nitrogen was separated in a mineral and organic fraction, with the organic N fraction for compost, digestate and scrubbed ammonium sulphate assumed to be 0%, 50% and 90%, respectively. Mineral N and organic N were assumed to have an N use efficiency of 80% and 55%, respectively. Phosphorus and potassium presented in organic materials were assumed to have a mineral fertiliser substitution efficiency of 85% and 73% (Eghball et al., 2002; Tonini et al., 2019). While applying the same principles to the case of domestic compost, it was assumed that only a fraction of the domestically produced compost substituted mineral fertilisers. Such correction factor was based on the findings of a survey published in Andersen et al. (2010) providing the fraction of domestic compost users that actually substituted mineral fertiliser (19-39%) in two Northern EU metropolitan areas. The average was used in the default calculation. Use of aged bottom ash as road sub-base filling material was assumed to substitute for natural gravel extraction and production, on a one-to-one mass basis following the approach of Birgisdottir et al. (2007).

### 2.5 Uncertainty analyses

We addressed uncertainty at two levels: i) by propagating the parameter uncertainty on the results through Montecarlo simulations and ii) by assessing the scenario uncertainty with respect to key scenario assumptions that we call now onwards as "default". With respect to the first, a triangular distribution was used for most parameters in line with the approach of Tonini et al. (2019). The range was based on primary information for the most sensitive and important parameters based on similar LCA studies (15% of total parameters, e.g. energy efficiency of incinerators and gas engines, transport distance, biogas yield, accidents variation over years, costs; see Tonini et al., 2019 and Bisinella et al. 2016); while a range equal to ±20% around the default value was assumed for the remaining (85% of total parameters). The main aim of propagating the parametrical uncertainty is to perform a robust MCDA to obtain scenario rankings supported by a discernibility analysis, i.e. deriving the number of occurrences when one scenario ranks best or better than the others. With respect to the scenario uncertainty, we assessed two key variants regarding energy and amendment market: i) the performance of the six scenarios under an energy system variant with a high penetration of natural gas representing the current Dutch electricity system in place of the marginal mix derived after European Commission (2016) used as default (energy variant); and ii) the performance under the assumption that the produced organic amendments are absorbed by the horticulture sector displacing the supply and use of peat (market variant; this means that compost is considered to be also a carbon source additionally to a nutrient source). To do this, we followed the approach suggested by Boldrin et al. (2009) and Andersen et al. (2010) assuming that the substitution of peat occurs on a volume basis, i.e. 1 kg of compost displaces 0.285 kg of peat and 1 kg of biochar displaces 1 kg of peat (density peat and biochar 0.2 kg m^−3^; density compost 0.7 kg m^−3^). For the case of domestic compost the correction factor suggested by Andersen et al. (2010) was further applied (i.e. only 19-22% of domestic compost produced actually substitutes for peat; the average was used in the default calculation).

## 3 Inventory data

We gathered local primary data to describe the *foreground system* bearing in mind the framework from Taelman et al., 2019: i) food waste composition, ii) separately and non-separately collected food waste flows (i.e. SC-FW and NSC-FW), iii) waste treatment technologies (i.e. input-output data related to material and energy use, emissions, product-outputs, labour and cost) and iv) other local/social information, e.g. accidents rate, location and land occupation of facilities/containers, distances households-to-waste containers, stakeholder participation in the scenarios proposal, and waste fees. Particular attention was devoted to detail local collection schemes with respect to spatial information for both the reference and the proposed alternative scenarios (Appendix B). All data retrieved referred consistently to the year 2015. Data on stakeholder involvement in the definition of the scenarios were recorded during the different project meetings and used for the calculation of the related indicator (Amenta *et al.*, 2019; Appendix B). Calculation details for the indicators Public Acceptance, Accessibility to WMS, and Landscape Disamenities are thoroughly reported in Appendix B with results reported in Appendix C (Table C.18-C.26). Accident rates in the waste management and transport sectors were retrieved from local databases (FEDRIS 2019; year 2015 used as default). *Background data* for the modelling of electricity, heat, materials, fuels and the provision of other resources was taken from the ecoinvent database 3.5 consequential system (Ecoinvent centre, 2019).

### 3.1 Food waste: quantity and composition

The total amount of food waste generated by households and SMEs in the AMA was quantified to 153,310 t food waste per year, wet weight (Appendix C; Table C.1). This is the same across all scenarios investigated. The composition of the food waste generated by households in the AMA was originally derived from primary data as disaggregated food macro-categories (e.g. meat, fruit, vegetables; Appendix A and C; Table C.3). For modelling purposes, these categories were further approximated to describe the physical-chemical composition of the foods using *ad hoc* food product-specific datasets as provided in recent publications (Tonini, et al., 2018; Appendix C, Table C.4). To obtain this products breakdown we followed the procedure described in Laurentiis et al. (2018) applying diet patterns specific to the Netherlands as reported in EFSA, (2015). The food waste composition is the same across all scenarios as the management scheme does not affect the type of food discarded by the households. To model the content of impurities (i.e. non-food waste material fractions such as plastic, paper, glass, and metals) in the separately collected food waste flow, we relied on the figures reported in Puig-ventosa et al. (2013). Based on this, "door-to-door" was assumed to incur a share of impurities equal to 6.8% while "bring” schemes to 16.2% (see description in section 3.3.1). The assumption here is that such impurity fractions are recyclable and should neither end up in the food nor in the mixed waste stream. Thus, they constitute impurities to be added to the annual food waste flow in both cases (see Appendix A for calculations and Appendix C, Table C.1 for results). The chemical composition of these materials was taken from Riber et al. (2009) (Table C.5).

### 3.2 Data and assumptions specific to the reference scenario

The information regarding the *status quo* of the food waste collection in the AMA is provided by the Dutch National Register of Waste (Ministry of Infrastructure and Water Management, 2019). The data includes all actors in the waste chain (waste generators, collectors, merchants, processors, etc.), their company details, quantity and type of waste, and treatment processes. Based on this, the distribution of the waste across the individual treatments is reported in Appendix C, Table C.2. The life cycle inventory for each individual technology involved in the reference scenario was compiled based on the information available from different sources. While primary data collection from the operators involved was prioritised (e.g. through specific documentations available on-line or direct contact), some of the data were also collected from scientific literature when primary data was lacking. Data regarding waste collection were obtained at the level of wastesheds (six in total; Figure 1b), prior to derive the average for the whole AMA; see Appendix A and Table C.6 for additional details. The data on the current distances from the households to the drop-off points are reported in Appendix C, Table C.18. The inventory data for collection, incineration, centralised composting, anaerobic digestion, biogas upgrading and post-composting plants can be found in Appendix C, Table C.8-to-C.11. The compost produced after biological treatment was assumed to be transported 25 km and locally applied on-land. Emissions from use-on-land (i.e. metal deposition on soil, leaching of N and P, air emissions of NH_3_, N_2_O, and biogenic CO_2_ sequestration during the considered 100-year time horizon after application) were quantified conforming to the modelling principles detailed in Tonini et al. (2019). Based on this, the emission of N_2_O-N, NH_3_-N, and NO_3_-N from compost equalled 1.0%, 0.9%, and 25% of the N applied. Leaching of P was calculated as the difference between the P applied and the P bioavailable to plants assuming no storage occurs (i.e. P-saturated soil reflecting current situation in the Netherlands; Tonini et al., 2019), equalling 23.5% of the P applied. Long-term carbon sequestration, within the 100y time horizon considered, was 10% of the C applied. Applying the same principles, the N_2_O-N, NH_3_-N and NO_3_-N emissions from concentrated N fertilisers (ammonium sulphate and mineral N fertilisers) equalled 1.0%, 0.9% and 10% of the N applied, respectively. Leaching from mineral P was calculated as 5% of the P applied. For thermal treatment residues, bottom ash was assumed to be aged, transported on average 100 km and used as aggregates for road pavement (sub-base) while fly ash was assumed to be transported on average 500 km and utilised as backfilling material in old salt mines in Germany conforming to the approach of Fruergaard et al. (2010).

### 3.3 Data and assumptions specific to the alternative scenarios

#### 3.3.1 Proposed changes in food waste collection schemes

An improved food waste collection system was designed, consisting of four major changes:

I) A "bring" food waste collection scheme in the centre of Amsterdam (“AEB” wasteshed; Figure 1b), using floating containers, is proposed for households that do not have access to a garden and for SMEs. Food waste is collected from these points. Outside the city centre (remaining wastesheds; Figure 1b), accessibility to the food waste collection system is increased by decreasing the number of households per container and collection point to the level of current mixed waste collection.
II) "Door-to-door" food waste collection is implemented in high-density areas in all the remaining wastesheds. Areas with more than 5,000 inhabitants per square km, which is a high density in the Netherlands, have door-to-door collection for both households with and without access to a garden. This means that every apartment building, row house, semi-detached house and single-family house have one container where all households and SMEs in the building dispose their food waste. In these areas, SMEs and households without access to a garden have one collection point per building, whereas households with access to a garden have one collection point every four households. Notice that this change does not apply to scenario *MBT*.
III) In the remaining wastesheds other than “AEB”, where population density is lower than 5,000 inhabitants per square km, accessibility to food waste collection systems is increased by decreasing the number of households per container and collection point to the level of current mixed waste collection ("bring" scheme). More details on the modelling may be found in Appendix B, while a summary of the spatial distribution of containers and collection points may be found in Appendix C, Table C.20. The related distance households-to-containers and calculation of the accessibility indicator may be found at Table C.21-C.22, Appendix C.
IV) To enhance participation in areas served with door-to-door collection, the fee for food waste separately collected is set to zero and costs are instead allocated to the mixed waste flow following best practices in EU (BiPRO/CRI, 2015; Appendix B).

#### 3.3.2 Waste treatments and processes

Incineration of the NSC-FW was assumed to occur at the local plant named AEB (capacity 1.4 Mt a^−1^; Appendix C, Table C.9) as no additional incineration capacity is required to treat the NSC-FW. The inventory for home composting (*hCP*) was based on Andersen et al. (2011). The inventory for the centralised composting and anaerobic digestion plant were assumed as those of existing facilities in Middenmeer (composting capacity 79,000 t a^−1^; digestion capacity 118,000 t a^−1^; see Table C.10-C.11). These choices are supported by the fact that comparable plants are required to treat the amount of separately collected food waste in these scenarios. The inventory for the production of ammonium sulphate from digestate and of biochar from compost (scenario *cAD-PP*) was based on, respectively, the stripping technology described in Errico et al., (2018) and the pyrolysis plant detailed in Tonini et al. (2019). The enzymes-based MBT to recover biomass from mixed waste was based on the technology described in Tonini et al. (2013). Enzymes are used to liquefy the biomass fraction of mixed MSW, i.e. food and paper, generating a bioliquid and a residual solid fraction (non-degraded materials) that is sent to incineration. For inventory details, the reader is referred to Appendix C, Table C.12-to-C.14. Biogas upgrading, compost use-on-land and fate of incineration residues were modelled similarly to the reference (section 3.2). Biochar was modelled similarly to compost, but assuming a carbon sequestration equal to 90% of the C applied with the product based on literature (Tonini et al., 2019), while ammonium sulphate was modelled as mineral N fertilisers.

## 4 Results

### 4.1 Breakdown of the impact assessment results per Area-of-Protection

**Figure 2:**
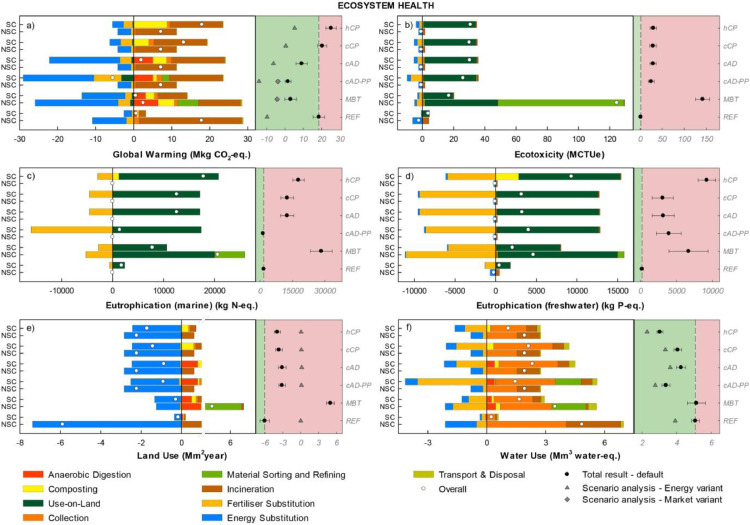
Disaggregated life-cycle results for the Area-of-Protection ecosystem health (FU: 153 kt food waste annually generated). Positive values indicate burdens, while negative indicate savings. The left-hand side of each graph illustrates the impacts for the treatment of SC-FW and NSC-FW in each scenario. The right-hand side of each graph illustrates the total scenario result (SC-FW + NSC-FW) for the default calculation (mean and error bars indicating plus minus one standard deviation) and for the system variants when applicable. Scenarios associated with a net saving relative to *REF* are displayed on the green background while those associated with a net burden relative to *REF* are displayed on the red background. “Anaerobic Digestion” represents all processes involved at the anaerobic digestion plant including pre-treatment of the waste, upgrading of biogas and dewatering of digestate; ”Collection” represents all operations of waste collection; “Composting” represents all processes involved at the composting plant including pre-treatment of the waste; “Incineration" represents all processes involved during incineration of the waste; “Material Sorting and Refining” includes the remaining mechanical, biological and chemical post-collection processes aiming to further sort the waste and recover materials (i.e. mechanical-biological treatment, pyrolysis, and ammonium sulphate stripping); “Fertilisers Substitution” represents savings from substitution of market mineral fertilisers with fertilisers derived from secondary raw material; “Energy Substitution” represents savings from substitution of market electricity, heat, and natural gas; “Use-on-Land” represent all processes involved in the application on-land of fertilisers derived from secondary raw material (operations and emissions, e.g. nutrient leaching and metals deposition following spreading on agricultural soil); "Overall" is the net impact, as sum of burdens and savings for the separately and non-separately collected food waste (SC-FW and NSC FW); "Total result - default" (black circle) represents the total impact result as sum of the impact associated with SC-FW and NSC-FW, while "Scenario analysis - energy system" (grey triangle) indicates the total impact result under the natural gas-based energy system variant.

**Figure 3:**
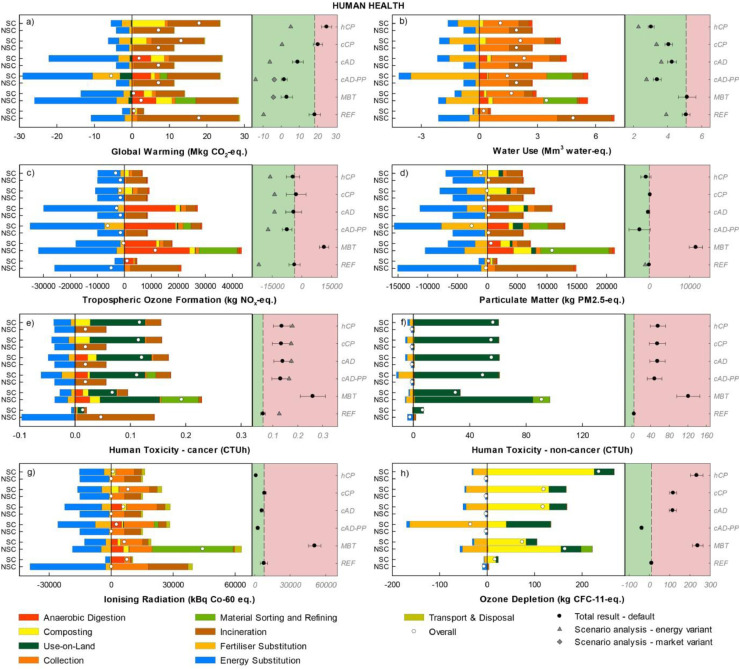
Disaggregated life-cycle results for the Area-of-Protection human health (FU: 153 kt food waste annually generated). Positive values indicate burdens, while negative indicate savings. The left-hand side of each graph illustrates the impacts for the treatment of SC-FW and NSC-FW in each scenario. The right-hand side of each graph illustrates the total scenario result (SC-FW + NSC-FW) for the default calculation (mean and error bars indicating plus minus one standard deviation) and for the energy and market variant when applicable. Scenarios associated with a net saving relative to *REF* are displayed on the green background while those associated with a net burden relative to *REF* are displayed on the red background. For the abbreviations and description of the legend refer to Figure 2.

**Figure 4:**
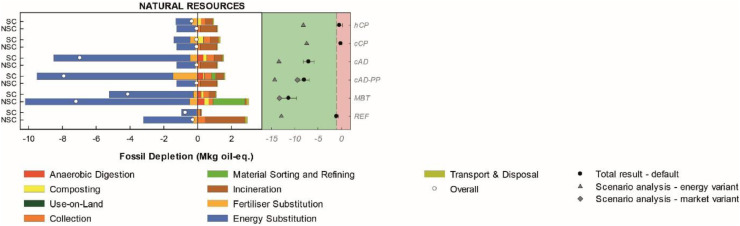
Disaggregated life-cycle results for the Area-of-Protection natural resources (FU: 153 kt food waste annually generated). Positive values indicate burdens, while negative indicate savings. The left-hand side of each graph illustrates the impacts for the treatment of SC-FW and NSC-FW in each scenario. The right-hand side of each graph illustrates the total scenario result (SC-FW + NSC-FW) for the default calculation (mean and error bars indicating plus minus one standard deviation) and for the energy and market variant when applicable. Scenarios associated with a net saving relative to *REF* are displayed on the green background while those associated with a net burden relative to *REF* are displayed on the red background. For the abbreviations and description of the legend refer to Figure 2.

**Figure 5:**
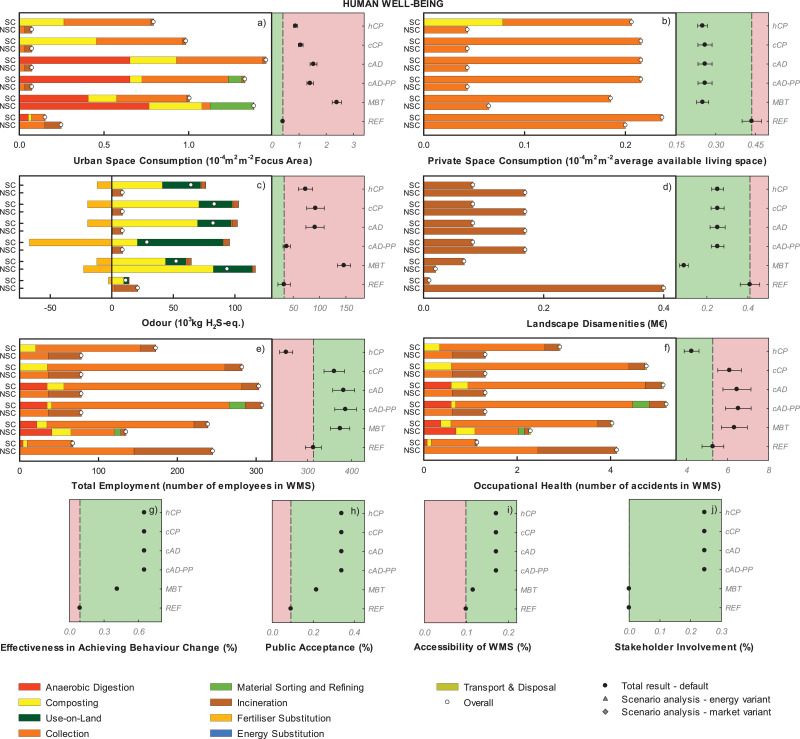
Disaggregated life-cycle results for the Area-of-Protection human well-being (FU: 153 kt food waste annually generated). Positive values indicate burdens (or increases, e.g. for Total Employment and the other social indicators), while negative indicate savings. The left-hand side of each graph illustrates the impacts for the treatment of SC-FW and NSC-FW in each scenario. The right-hand side of each graph illustrates the total scenario result (SC-FW + NSC-FW) for the default calculation (mean and error bars indicating plus minus one standard deviation) and for the energy and market variant when applicable. Scenarios associated with a net saving relative to *REF* are displayed on the green background while those associated with a net burden relative to *REF* are displayed on the red background. For the abbreviations and description of the legend refer to Figure 2.

**Figure 6:**
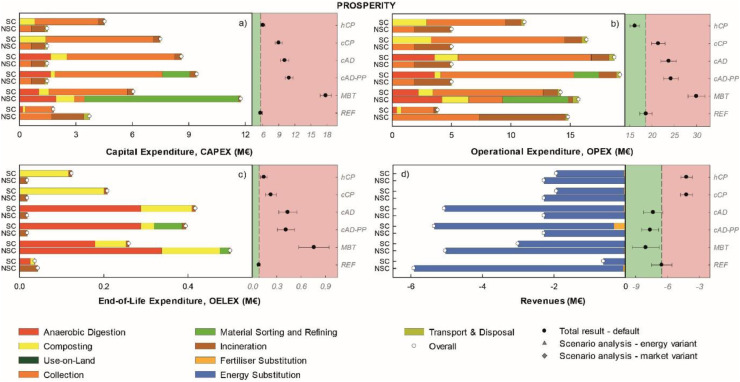
Disaggregated life-cycle results for the Area-of-Protection prosperity (FU: 153 kt food waste annually generated). Positive values indicate burdens, while negative indicate savings (i.e. here revenues). The left-hand side of each graph illustrates the impacts for the treatment of SC-FW and NSC-FW in each scenario. The right-hand side of each graph illustrates the total scenario result (SC-FW + NSC-FW) for the default calculation (mean and error bars indicating plus minus one standard deviation) and for the energy and market variant when applicable. Scenarios associated with a net saving relative to *REF* are displayed on the green background while those associated with a net burden relative to *REF* are displayed on the red background. For the abbreviations and description of the legend refer to Figure 2.

The disaggregated impacts per impact category are displayed in Figure 2 - 6 grouped according to each AoP. Positive values indicate burdens (or increases e.g. for Total Employment, Occupational health, and the other social indicators), while negative indicate savings. The left-hand side of each graph illustrates the impact for the treatment of SC-FW and NSC-FW in each scenario. The right-hand side of each graph illustrates the total result per scenario (with dots; sum of the impacts associated with SC-FW and NSC-FW) compared to the reference scenario (mean and error bars indicating plus/minus one standard deviation); the results for the energy and market variant are reported when they lie outside the parameter uncertainty bar.

#### 4.1.1 Area-of-Protection ecosystem health

Under the default assumptions, scenario *REF* performed best in three out of six midpoint indicators, namely for Ecotoxicity, Freshwater Eutrophication, and Land Occupation (Figure 2). While the reason for the latter was the greater energy substitution from incineration, for the remaining indicators the better performance was due to the reduced leaching of nutrients and return of metals to agricultural soil following incineration of the food waste and disposal compared to the alternative scenarios. In these, instead, nutrients and residual organic biomass are mostly returned to cropland: while a displacement effect was obtained by substituting conventional mineral fertiliser production and their use-on-land (including the associated leaching effects), this was nevertheless not sufficient to compensate for the induced burdens.

In the category Global Warming, *cAD-PP* performed best followed by *MBT* (Figure 2a). *cAD-PP* achieved larger benefits compared to the remaining scenarios thanks to the combination of gas recovery (energy substitution), increased carbon sequestration through biochar and greater substitution of mineral fertilisers (displayed under the stack "Use-on-Land"; Figure 2a). The reason for this was the improved N plant bioavailability achieved with the production of ammonium sulphate, a product that has a more efficient plant uptake. Consequently, N-leaching from use-on-land was reduced, incurring a better performance in Eutrophication (marine). This was not the case for Eutrophication (freshwater), where *REF* performed better owing to the lower amount of phosphorous returned to agricultural soil. It should be noticed that nutrient leaching from road sub-base aggregates, while accounted for, is typically negligible. Among all scenarios, home composting (*hCP*) showed the lowest consumption of water owing to the reduced waste collection operations (Figure 2f). For the scenario *MBT*, an important contribution to the impact on Ecotoxicity, Marine Eutrophication, Land Occupation and Water Consumption (Figure 2b,c,e,f) was associated to the use of enzymes in the biomass recovery and separation process (see the stack "Material Sorting and Refining" in Figure 2). Notice that for *MBT*, impacts/savings are partially shifted from SC-FW to NSC-FW because a substantial portion of the food waste was not separated at the source, ending up in the mixed waste treatment scheme (NSC-FW).

Considering the energy variant, the main difference observed is a change in the ranking of the scenarios for Global Warming and Land Use. For the former, only the scenario *cAD-PP* performed better than the *REF,* for which energy substitution savings are increased under a natural gas-based energy system. For Land Use, the differences observed across scenarios became negligible. Significant uncertainties were observed for *MBT* in the categories Ecotoxicity and Eutrophication, mainly associated with the amount of enzymes used.

#### 4.1.2 Area-of-Protection human health

Under the default assumptions, *REF* performed best in two out of eight indicators, namely Human Toxicity cancer and non-cancer (Figure 3e,f). For these, *REF* incurred lower impacts from use-on-land, due to a lower contribution from metals return to agricultural soil, as observed earlier for Ecotoxicity under the AoP ecosystem health. For *hCP*, fugitive emissions during home composting, particularly N_2_O and CH_4_, alongside the poor energy and fertiliser substitution effect, negatively affected the performance in Global Warming and Ozone Depletion. The scenario *cAD-PP* performed best in Global Warming, Tropospheric Ozone Formation, Particulate Matter, and Ozone Depletion thanks to the higher displacement of mineral N fertilisers' production by ammonium sulphate relative to the remaining scenarios including composting. The scenario *MBT*, while achieving the second best performance in Global Warming, incurred the worst performance in the toxicity categories mainly due to the overall larger return of organic material and metals to agricultural soils, and in Tropospheric Ozone Formation, Particulate Matter, Ionising Radiation and Ozone Depletion mainly because of the impacts from mechanical-biological processing and enzymes used. Under the energy variant, the main effect observed was the change in the ranking of the scenarios in Tropospheric Ozone Formation, where *REF* performed best because of the larger fossil fuel substitution effect obtained from energy recovery. As in the AoP ecosystem health, significant uncertainties were observed for *MBT* and, in general, for the results in the toxicity impact categories for all scenarios.

#### 4.1.3 Area-of-Protection natural resources

Under the default assumptions, *MBT* performed best in the category fossil resource depletion, followed by *cAD-PP*, while home and centralised composting scenarios (*hCP* and *cCP*) performed worst (Figure 4). The savings from energy substitution were the most important contributions, followed by the burdens associated with the different waste treatment operations involved, notably incineration, collection and additional operations of sorting and refining. The ranking of the scenarios changed under the energy variant, in which *cAD-PP* was best, followed by c*AD* and *REF*. This was due to a greater energy substitution effect obtained from incineration of the NSC-FW flow. Such variant affects minimally *MBT,* as most food waste is processed into natural-gas quality with a negligible share undergoing incineration. Resource-intensive sorting and refining operations in *cAD-PP* and *MBT* were mainly caused by sulphuric acid and enzymes consumptions, respectively, which were assumed to be produced and supplied by the global market and thus not dependent on the variations of the local energy system.

#### 4.1.4 Area-of-Protection human well-being

The total urban space consumption increased by a factor three-to-six when comparing *REF* to the alternative scenarios (Figure 5a); the scenario *MBT* showed the highest footprint in terms of urban space consumption due to the increased land requirements for anaerobic digestion and composting; considering the uncertainties, private space consumption was similar for the alternative scenarios assessed (Figure 5b). In the odour footprint, *REF* (Figure 5c) and *cAD-PP* performed best because of the reduced ammonia emissions during use-on-land and composting compared to the remaining scenarios. For disamenities, all scenarios achieved a reduction of the impact compared to *REF,* owing to the reduced amount of waste sent to incineration, here the main cause of property value loss. *MBT* achieved the best performance as in this scenario the amount of waste incinerated was the least across all scenarios (Figure 5d). The number of employees was highest for the scenarios involving a maximum of separate collection and post-processing operations, i.e. *MBT, cAD-PP*, and *cAD* while *REF* held the lowest (Figure 5e). Accidents were correlated to employment, and thus higher for the scenarios having a greater number of employees (Figure 5f). All the remaining social indicators (Effectiveness in Achieving a Behaviour Change, Public Acceptance, Accessibility and Stakeholder Involvement) were highest for the scenarios involving a maximum of food waste separate collection (Figure 5g,h,i,j). Important uncertainties were observed for Private Space Consumption, Odour, Total Employment and Occupational Health. No changes are expected under the energy and market variant.

#### 4.1.5 Area-of-Protection prosperity

*MBT* incurred the highest capital and operational costs followed by *cAD-PP* and c*AD* (Figure 6a,b). *REF* was always the cheapest solution across all cost indicators, followed by *hCP*. Across all scenarios, (separate) collection was by far the largest contribution to the overall impact in CAPEX and OPEX (Figure 6a,b). The capital cost of the MBT-plant also represented a significant contribution to the CAPEX (Figure 6a), as a significant portion of the food waste generated was treated via MBT without prior separation. End-of-Life Expenditures (OELEX; Figure 6c) were about one order of magnitude smaller than CAPEX and OPEX, and generally showed the same pattern as for CAPEX; being in general correlated to the size of the facilities to be dismantled, with composting and anaerobic digestion as the major contributions. *MBT* achieved the largest revenues followed by *cAD-PP* and *cAD* (Figure 6d). Home and centralised composting (*hCP* and *cCP*) incurred the lowest revenues among all; these came from incineration of non-separately collected food waste and rejects. While energy substitution (gas and electricity, mainly) was the major source of revenues across all scenarios, for the specific case of *cAD-PP,* the revenues from selling of N-fertiliser and soil amendments were significantly increased compared to the remaining scenarios. Under the market variant the revenues from selling amending products were increased in all scenarios but did not affect the overall ranking. No changes are expected under the energy variant, as we did not assume variations in the energy price.

### 4.2 Ranking of the scenarios after multi-criteria decision analysis

Under the default assumptions and considering the results of parameter uncertainty propagation, *cAD-PP* ranked best in two out of five AoPs, i.e. ranked first in 74% of the occurrences in AoP ecosystem health and in 98% of the occurrences in AoP human health. Home composting (*hCP*) ranked best in 61% of the occurrences in AoP human well-being. *REF* ranked best in 85% of the occurrences in AoP ecosystem health and in 100% of the occurrences in AoP prosperity (Table 1). The scenario *MBT* ranked first in 93% of the occurrences in AoP natural resources. All in all, *cAD-PP* performed better than *REF* in three out of five AoPs and comparable in one out of five; *cAD* in three out of five, *MBT* in two out of five and *hCP/cCP* only in one out of five. These rankings were not significantly affected under the market and energy variants; for the latter, the main change observed was that *cAD-PP* ranked best also in the AoP natural resources. This was due to the larger savings of fossil fuel resource obtained from incineration of the NSC-FW flow under a natural gas-based energy system compared to the low-carbon used as default.

**Table 1:** Results of the multi-criteria decision analysis (MCDA) for each Area-of-Protection for the default calculations and the two scenario variants. The number indicates the ranking achieved after applying the MCDA, with 1 indicating the best and 6 the worst scenario. The percentage indicates the number of occurences in which the scenario ranked first, based on Montecarlo (1000 simulations). c*AD*: centralised anaerobic digestion*; cAD-PP*: centralised anaerobic digestion with post-processing of the digestate; *cCP*: centralised composting; EH: ecosystem health; *hCP:* home composting; HH: human health; HW: human well-being; *MBT*: mechanical-biological treatment; NR: natural resource; PR: prosperity. *REF*: reference scenario. Notice that two scenarios may have the same rank because of the pairwise nature of MCDA (this is also reflected in the number of occurrences).

	**Default**					**Energy variant**					**Market variant**				
	**EH**	**HH**	**HW**	**NR**	**PR**	**EH**	**HH**	**HW**	**NR**	**PR**	**EH**	**HH**	**HW**	**NR**	**PR**
*hCP*	5	5	1|61%	5	2	5	5	1	5	2	5	5	1	6	2
*cCP*	3	4	3	6	5	3	4	3	6	5	3	4	3	5	5
*cAD*	3	2	4	3	2	3	3	4	2	2	3	2	4	3	3
*cAD-PP*	1|74%	1|98%	2	2	4	1	1	1	1	4	1	1	2	2	3
*MBT*	6	6	4	1|93%	5	6	6	4	4	5	6	6	4	1	5
*REF*	1|85%	3	6	4	1|100%	1	2	6	3	1	1	3	6	4	1

## 5 Discussion

### 5.1 Concrete learnings and recommendations for the focus area

Amid the scenarios assessed, we identify anaerobic digestion with effective energy and nutrient recovery as an option capable to improve the overall sustainability of the current management system in all the Areas-of-Protection assessed but prosperity. While common ‘circular’ food waste management options rely on composting and anaerobic digestion, the actual market value of compost and digestate is rather low; in the Netherlands the market prices can be even negative, ranging from -5 to 2 € kg^−1^ wet weight based on information from local operators. Because of the low nutrient concentration, but high organic carbon content, compost and digestate are often used as soil improvers rather than fertilisers. In spite of the added value that organic matter could possibly have for agriculture under specific conditions, the return on investment from applying these materials on agricultural land varies substantially depending on local conditions (Hijbeek et al., 2017), with the strongest effects likely to be observed in the long-term. Therefore, farmers may be reluctant to buy and apply compost and digestates; as a matter of fact, they are frequently paid to do so (Gebrezgabher et al., 2010; Huygens et al., 2019). Considering the need to transport nutrients from the urban to the rural system, opportunities are present to produce nutrient-dense fertilisers with comparable agronomic properties as their mineral alternative or to manufacture specific niche products like biochar. The latter can, for instance, serve as more sustainable alternatives to existing products such as peat (Margenot et al., 2018; Velthof, 2015). Although the added value of biochar in an European agricultural context remains disputed (Jeffery et al., 2017), opportunities to apply biochar exist in diverse areas and niche sectors (Carlile et al., 2019).

As for all the alternative scenarios assessed, the scenario with digestion and advanced post-processing of the digestate is associated with higher costs compared to the reference (mainly based on incineration without source-separation) because of the increased collection expenses. This option, however, offers advantages in most environmental and social impact categories thanks to the benefits derived from energy substitution, reduced nutrient leaching and metals deposition on agricultural soil. The added value of the post-processing lies in the production of a more efficient N-fertiliser next to the niche product (biochar), both having a possible higher local demand than digestate or compost. Under the conditions assumed for the area under study, schemes based on home and centralised composting are associated to more adverse impacts, especially for human health and natural resources but also for prosperity in the case of centralised compositing owing to the poor revenues and high collection costs. The centralised mechanical-biological option performed best in fossil resource depletion (Figure 4) but was affected by the impact related to enzymes supply in the remaining impact categories and by overall high costs. Two main criticalities were observed for this scenario: firstly, significant uncertainty is connected to the consumption of enzymes and its dataset, which calls for further investigations. Secondly, food waste source separation should be enforced to conform with the EU Waste Framework Directive (European Parliament and Council, 2018) and the use-on-land of food waste-derived fertilisers is typically allowed with the pre-condition that the food waste is separated at the source (European Parliament and Council, 2019). While this assessment indicates clear priorities for the ideal management scheme, an aspect calling for further investigation regards the portfolio of technical and socio-economic tools to increase separate collection rates, here assumed to be finally in line with the EU 2030 goals. This calls for a different type of study, e.g. identification of best practices, and was beyond the scope of this analysis.

### 5.2 Transforming scattered data into a useful format for stakeholders

This article presents the application of a sustainability framework that can be used to bridge the gap between science and decision-making. The application is illustrated with a case study on different food waste management options for the Amsterdam Metropolitan Area. This study is part of a broader EU Horizon 2020 project namely REPAiR (Resource Management in Peri-Urban Areas), whose ambition is to shed new light on participatory and science-based decision-making by involving local stakeholders in the entire process. The method started by listing the most relevant impacts of waste management identified by a wide range of stakeholders and the literature, covering social, economic and environmental areas e.g. costs, urban space consumption, sustainable use of natural resources, local pollution and emissions, and legislative requirements. The approach applied relies on the input of the (local) stakeholders involved in order to collect, to the best possible extent, perspectives and interests of those parties affected by decisions. While data collection is often the bottleneck for the operationalisation of the sustainability frameworks proposed in literature, we strived to achieve a detailed representation of the local conditions. Dividing the area into six wastesheds, we collected detailed local data on food waste flows and composition alongside input-output data on collection and treatment technologies involved, spatial data such as urban and private land occupation for containers and technologies, accessibility of waste containers, market prices, waste fees, employment and accidents. In combination with the extensive focus on uncertainty analysis, the inventory data enabled detailed life cycle assessments, with the results as presented in Figure 2-to-6. While we applied state-of-the-art uncertainty analyses techniques, improvement margins still exist as primary data on uncertainties were available only for some key parameters. This said, the greatest impact is achieved when scientific results are presented in a manner that makes them accessible to the widest possible audience and stakeholders involved in the decision process. Scientific analysis and communication is adequate if it reaches people with the information they need in a form that they can use (Fischhoff and Bird, 2013). Therefore, results were aggregated through multi-criteria decision analysis in a simple overview (Table 1) summarising which scenario performed best per Area-of-Protection, a scientific analysis that is very relevant for decision-making. This enables a discussion that considers the synergies and trade-offs amongst environmental, economic and social impacts, to foster decisions that maximise overall societal benefits. In the first place, the goal of this analysis is to provide a transparent and clear understanding of the findings so that stakeholders can discuss based on their perspectives and value issues, such as how much weight to give to the different Areas-of-Protection. Hence, the primary objective of the user-oriented approach is not to achieve an overall agreement in a negotiation and decision process on waste management, but to have informed disagreements across stakeholders that have different needs and priorities (Fischhoff and Bird, 2013).

## 6 Conclusion

By quantifying the environmental, economic, and social impacts based on the priorities of local stakeholders, this study evaluates the sustainability of different options for the management of household food waste using the Amsterdam Metropolitan Area as a case study. Among the options assessed, anaerobic digestion coupled with effective nutrient and energy recovery appears to be the preferred option to improve the overall sustainability of the current system in all Areas-of-Protection but prosperity, where the *status quo* still performs better due to the overall lower costs. By collecting and processing multi-fold data, with a strong focus on site-specific information, into a comprehensive and broadly understandable framework, we provide local stakeholders and authorities with science-based evidence to support actions and policies in relation to household food waste. The results serve as a basis to prioritise sustainable solutions in the future waste and circular economy strategy specifically for Amsterdam, but could be useful as well in other European areas having similar characteristics.

## Declaration of Competing Interest

The authors declare that they have no known competing financial interests or personal relationships that could have appeared to influence the work reported in this paper.

## Appendix Supplementary materials

Image, application 1

Image, application 2

Image, application 3
