# Supplementary material for: Quantitative sustainability assessment of household food waste management in the Amsterdam Metropolitan Area
Source: Resour Conserv Recycl. 2020 Sep;160:104854. doi: 10.1016/j.resconrec.2020.104854 (PMC7323620; doi:10.1016/j.resconrec.2020.104854)
Supplement: Supplementary file 1 [file mmc1.docx]

**Appendix A**

**Supplementary Notes**

**Quantitative sustainability assessment of household food waste management in the Amsterdam Metropolitan Area**

Davide Tonini^1^*, Alex Wandl^2^, Kozmo Meister^2^, Pablo Muñoz Unceta^2^, Sue Ellen Taelman^3^, David Sanjuan-Delmas^3^, Jo Dewulf^3^, Dries Huygens^1^

^1^European Commission, Joint Research Centre (JRC), Seville, Spain

^2^Technical University of Delft, Delft, the Netherlands

^3^Ghent University, Faculty of Bioscience Engineering, Department of Green Chemistry and Technology, Sustainable Systems Engineering Group (STEN), Ghent, Belgium

This Supporting Information document contains additional information on the foreground and background data used in the study.

For all the related Tables the reader is referred to the accompanying Excel-file "Appendix C – Supplementary datasets".

[Mass and energy flows in the scenarios assessed 2](#_Toc27577858)

[Distribution of the food waste generated across treatments (reference scenario) 2](#_Toc27577859)

[Food waste and impurities composition 3](#_Toc27577860)

[Inventory for waste collection 5](#_Toc27577861)

[Inventory for incineration technology 6](#_Toc27577862)

[Inventory for anaerobic digestion technology 6](#_Toc27577863)

[Inventory for centralised composting and post-composting technology 6](#_Toc27577864)

[Inventory for mechanical-biological technology 7](#_Toc27577865)

[Inventory for ammonium sulphate production technology 7](#_Toc27577866)

[Inventory for biochar production technology 8](#_Toc27577867)

[Inventory for transport 8](#_Toc27577868)

[Background inventory data and unit-price for marketable goods 9](#_Toc27577869)

[References 10](#_Toc27577870)

## Mass and energy flows in the scenarios assessed

| **Supplementary information available** | |
| --- | --- |
| Table C.1 | Main mass and energy flows in the scenarios assessed, expressed per functional unit (food waste generated in the Focus Area during a year). Note that the flow of C in organic fertiliser does not consider the subsequent mineralization following use-on-land; ww: wet weight. Electricity output and associated credit are reported as gross value. Values are rounded. |
| **Explanatory notes** |  |
| **-** | |

## Distribution of the food waste generated across treatments (reference scenario)

| **Supplementary information available** | |
| --- | --- |
| Table C.2 | Distribution of the food waste generated between the treatment plants in the reference scenario (year 2015; reference scenario *REF*) management system. AMA: Amsterdam Metropolitan Area. |
| **Explanatory notes** |  |
| **-** | |
|  |  |

## Food waste and impurities composition

| **Supplementary information available** | |
| --- | --- |
| Table C.3 | Food waste fractional composition as modelled for the Focus Area (% of wet weight, ww). All the datasets used as a proxy for the modelling are taken from the database of food products provided in Tonini et al. (2018) unless otherwise specified. |
| Table C.4 | Chemical composition of 1000 kg wet weight (ww) of food waste as generated in the Focus Area. All the datasets used as a proxy for the modelling are taken from the database of food products provided in Tonini et al. (2018) unless otherwise specified. Cbio and: carbon biogenic anaerobically digestible based on the methane potential. CH4 pot: methane potential; Energy: energy content before deducting the energy for the evaporation of water; TS: total solids; VS: volatile solids. |
| Table C.5 | Chemical composition of 1000 kg wet weight (ww) of impurities as modelled for the Focus Area. All the datasets used as a proxy for the modelling are taken from Riber et al. (2009). C bio and: carbon biogenic anaerobically digestible based on the methane potential. CH4 pot: methane potential; Energy: energy content before deducting the energy for the evaporation of water; TS: total solids; VS: volatile solids. |
| Eqs. A.1-A.4 | - |
| **Explanatory notes** |  |
| The fractional composition of the food waste was first derived in terms of food macro-categories based on primary data (Table C.3; column A and B). These were then approximated using dedicated physico-chemical datasets for food products as provided in Tonini et al. (2018). The impurities were modelled taking the following assumptions:   1. The impurities consist of recyclables and should thus neither end up in the food nor in the mixed waste stream. Thus, they constitute impurities to be added to the annual food waste flow for both SC-FW and NSC-FW. 2. We base the content of impurities in door-to-door and bring schemes on the figures provided in Puig-ventosa et al. (2013): Door-to-door: 6.8% (2.8%-10.7%; 2006-2008) and bring schemes 16.2% (8.8%-23.5%; 2006-2008). 3. The portion of the Focus Area not served by separate collection (door-to-door or bring) incurs a level of impurities *I* in its mixed waste equal to the bring scheme (Equation A.2). 4. The material fraction composition of the impurities was modelled on the basis of the figures reported in Puig-ventosa et al. (2013) and using the physico-chemical datasets provided in Riber et al. (2009) to describe the properties of the material fractions.   The content of impurities (% of the sum of food waste plus impurites) can be calculated as:  $I_{SC-FW}={CV}_{door}\cdot I_{door}+{CV}_{bring}\cdot I_{bring}$  **Equation A.1**  $I_{NSC-FW}=\left[ {CV}_{door}\cdot I_{door}+{CV}_{bring}\cdot I_{bring}+(100\%-{CV}_{door}-{CV}_{bring})\cdot I_{bring} \right]$  **Equation A.2**  The mass flow of impurities (kg ww) can then be recalculated as:  ${IW}_{SC-FW}=(SC-FW)\cdot\frac{{(CV}_{door}\cdot I_{door}+{CV}_{bring}\cdot I_{bring})}{{1-(CV}_{door}\cdot I_{door}+{CV}_{bring}\cdot I_{bring})}$  **Equation A.3**  ${IW}_{NSC-FW}=(NSC-FW)\cdot\frac{\left[ {CV}_{door}\cdot I_{door}+{CV}_{bring}\cdot I_{bring}+(100\%-{CV}_{door}-{CV}_{bring})\cdot I_{bring} \right]}{1-\left[ {CV}_{door}\cdot I_{door}+{CV}_{bring}\cdot I_{bring}+(100\%-{CV}_{door}-{CV}_{bring})\cdot I_{bring} \right]}$  **Equation A.4**  *I_SC-FW_*: impurities in separately collected food waste (% of food waste plus impurites)  *I_NSC-FW_*: impurities in non-separately collected food waste (% of food waste plus impurites)  *CV_door_*: coverage of door-to-door (% of households in the FA served with door-to-door)  *CV_bring_*: coverage of bring (% of households in the FA served with a bring-in scheme)  *I_door_*: content of impurities assumed for door-to-door (% in the food waste flow collected)  *I_bring_*: content of impurities assumed for bring-in (% in the food waste flow collected)  *IW_SC-FW_*: impurities flow in separately collected food waste (kg ww a^-1^)  *IW_NSC-FW_*: impurities flow in non-separately collected food waste (kg ww a^-1^)  NSC-FW: flow of separately collected (pure) food waste (kg ww a^-1^)  SC-FW: flow of non separately collected (pure) food waste (kg ww a^-1^) | |
|  | |

## Inventory for waste collection

| **Supplementary information available** | |
| --- | --- |
| Table C.6 | Spatial data used to model the collection of food and mixed waste in the reference scenario (*REF*). AMA: Amsterdam Metropolitan Area; NSC-FW: non separately collected food waste; SC-FW: separately collected food waste. |
| Table C.7 | Spatial data used to model the proposed collection system of food and mixed waste in the scenarios I-to-V (*hCP, cCP, cAD, cAD-PP, MBT*). AMA: Amsterdam Metropolitan Area; NSC-FW: non separately collected food waste; SC-FW: separately collected food waste. |
| Table C.8 | a) Amortised annuities and b) one-off costs expressed per t collected associated with food waste separately and non-separately collected in the individual scenarios. NSC-FW: non-separately collected food waste; SC-FW: separately collected food waste. |
| **Explanatory notes** |  |
| The data for the food and mixed waste collection in the scenario *REF* were based on 2015 primary spatial information collected on the Focus Area (Table C.6). The data for the food waste collection in the alternative scenarios I-to-V (*hCP, cCP, cAD, cAD-PP,* and *MBT*; Table C.7) were based on sound assumptions as described in Appendix B - Supplementary Methods. The collection costs for the reference and alternative scenarios were then calculated following the modelling approach described in Martinez-Sanchez et al. (2015) (Table C.8). | |

## Inventory for incineration technology

| **Supplementary information available** | |
| --- | --- |
| Table C.9 | Input-output disaggregated inventory for the incineration plants treating the food waste generated in the Focus Area. CAPEX: capital expenditures; EOLEX: end-of-life expenditures; LHV_ww_: lower heating value, wet basis; ww: wet weight. Notice that when recalculating the cost per tonne of waste treated, the result is reported per tonne of MSW and per tonne of food waste (in brackets). Values are expressed on a wet weight basis unless otherwise stated. |
| **Explanatory notes** |  |
| Datasets for thermal conversion technologies were mostly based on primary data collected from operators unless otherwise specified. Transfer coefficients of metals to air and ashes were based on the findings of Astrup et al. (2011). Insurance and maintenance costs were based on Martinez-Sanchez et al. (2015). EOLEX were estimated using the approach provided in Homes and Communities Agencies (2015) (see Appendix B - Supplementary Methods for details). All costs were recalculated per tonne of input-waste treated using the methodology described in Martinez-Sanchez et al. (2015) (amortisation and annualisation of a cost). | |

## Inventory for anaerobic digestion technology

| **Supplementary information available** | |
| --- | --- |
| Table C.10 | Input-output disaggregated inventory for anaerobic digestion, including biogas upgrading unit. AD: anaerobic digestion; CAPEX: capital expenditures; EOLEX: end-of-life expenditures; PF: plug flow. Values are expressed on a wet weight basis unless otherwise stated. |
| **Explanatory notes** |  |
| Datasets for anaerobic digestion plants were based on a mix of primary data collected from operators and literature. Data for operations and emissions were based on Boldrin et al. (2011). Insurance and maintenance costs were based on Martinez-Sanchez et al. (2015). EOLEX were estimated using the approach provided in Homes and Communities Agencies (2015) (see Appendix B - Supplementary Methods for details). All costs were recalculated per tonne of input-waste treated using the methodology described in Martinez-Sanchez et al. (2015) (amortisation and annualisation of a cost). | |

## Inventory for centralised composting and post-composting technology

| **Supplementary information available** | |
| --- | --- |
| Table C.11 | Input-output disaggregated inventory for composting. CAPEX: capital expenditures; EOLEX: end-of-life expenditures; GECO and GICOM are the names of the operators; PACOM: Process-based Aerobic composting. Values are expressed on a wet weight basis unless otherwise stated. |
| **Explanatory notes** |  |
| For composting, a mix of primary data and literature was used. Data for operations and emissions were based on Boldrin et al. (2011). Insurance and maintenance costs were based on Martinez-Sanchez et al. (2015). EOLEX were estimated using the approach provided in Homes and Communities Agencies (2015) (see Appendix B - Supplementary Methods for details). All costs were recalculated per tonne of input-waste treated using the methodology described in Martinez-Sanchez et al. (2015) (amortisation and annualisation of a future cost). Inventory for mechanical-biological technology  \| **Supplementary information available** \| \| \| --- \| --- \| \| Table C.12 \| Input-output disaggregated inventory for the enzymes-based MBT plant. CAPEX: capital expenditures; EOLEX: end-of-life expenditures. Values are expressed on a wet weight basis, unless otherwise stated. \| \| **Explanatory notes** \| \| \| Data for the enzymes based technology are based on Tonini et al. (2014), (2013). Detailed information on the technology may be found in Tonini et al. (2014) , (2013) and DONG (2015). EOLEX were estimated using the approach provided in Homes and Communities Agencies (2015) (see Appendix B - Supplementary Methods for details). \| \| | |

## Inventory for ammonium sulphate production technology

| **Supplementary information available** | |
| --- | --- |
| Table C.13 | Input-output disaggregated inventory for the stripping unit producing ammonium sulphate. CAPEX: capital expenditures; EOLEX: end-of-life expenditures. Values are expressed on a wet weight basis unless otherwise stated. |
| **Explanatory notes** |  |
| The inventory was compiled mainly using the data reported in Errico et al. (2018). Based on this, we assumed an annual capacity of 216,000 t of dewatered digestate (75% water content) and an efficiency of N recovery as N in (NH_4_)_2_SO_4_ equal to 95% of the input-N in the digestate, which is also in agreement with Tampio et al. (2016). Energy and chemicals consumptions (Errico et al., 2018) equalled (expressed per kg input-N): electricity 2 kWh, H_2_SO_4_ 1.82 kg, NaOH 0.54 kg, heat 12,3 MJ. CAPEX was 4.9 M€ while maintenance and insurance were assumed to equal 3% and 1.5% of the CAPEX as for anaerobic digestion. EOLEX were estimated using the approach provided in Homes and Communities Agencies (2015) (see Appendix B - Supplementary Methods for details). | |

## Inventory for biochar production technology

| **Supplementary information available** | |
| --- | --- |
| Table C.14 | Input-output disaggregated inventory for the pyrolysis unit producing biochar. CAPEX: capital expenditures; EOLEX: end-of-life expenditures. Values are expressed on a wet weight basis unless otherwise stated. |
| **Explanatory notes** |  |
| Details on the technology may be found in Huygens et al. (2019) and Tonini et al. (2019). EOLEX were estimated using the approach provided in Homes and Communities Agencies (2015) (see Appendix B - Supplementary Methods for details). | |

## Inventory for transport

| **Supplementary information available** | |
| --- | --- |
| - |  |
| **Explanatory notes** |  |
| Transport was assumed to occur with a truck 28-32 t, consuming 2.48E-05 kg diesel km^-1^ with a usage rate of 27,000 t y^-1^ and 6a lifetime. The initial investment cost (CAPEX) was assumed to be equal to 276,000 €. Maintenance and insurance were assumed to equal 7% and 1.4% of the CAPEX based on Martinez-Sanchez et al. (2015). This translates into a cost, per tonne transported, equal to 10.3 € t^-1^ (2.9 € for the sum of CAPEX, maintenance and insurance, 5.7 € for labour, 1.7 € for fuel). EOLEX were disregarded. The transport distances assumed were:   - Transport of compost or other organic fertilisers to use-on-land: 50km - Transport of solid fraction from MBT to incineration: 100km - Transport of bottom ashes to use as road base: 500km - Transport of fly ash to disposal: 500km   An uncertainty propagation analysis was carried out to test the sensitivity of these assumptions on the results. | |

## Background inventory data and unit-price for marketable goods

| **Supplementary information available** | |
| --- | --- |
| Table C.15 | Unit-cost for the marketable goods used in the inventory of the assessment. Amounts are expressed on a wet mass basis unless otherwise stated. |
| **Explanatory notes** |  |
| Background data for chemicals, materials, energy, and fuels used as input to the foreground system (i.e. technologies and processes) were mainly retrieved from the Ecoinvent v3.5 database, consequential system version (market processes). When not available, alternative datasets were used. Unit-costs, here reported as factor prices, were derived from various sources (see Table C.15). | |

## References

Astrup, T., Riber, C., Pedersen, A.J., 2011. Incinerator performance: effects of changes in waste input and furnace operation on air emissions and residues. Waste Manag. Res. 29, 57–68.

Boldrin, A., Neidel, T.L., Damgaard, A., Bhander, G.S., MÃ¸ller, J., Christensen, T.H., 2011. Modelling of environmental impacts from biological treatment of organic municipal waste in EASEWASTE. Waste Manag. 31, 619–630.

DONG, 2015. REnescience Northwich Appendix F : Application Site Condition Report.

Errico, M., Fjerbaek, L., Anne, S., Nielsen, K., Norddahl, B., 2018. Treatment costs of ammonia recovery from biogas digestate by air stripping analyzed by process simulation 1479–1489.

Homes and Communities Agencies, 2015. Guidance on dereliction , demolition and remediation costs.

Huygens, D., Saveyn, H., Tonini, D., Eder, P., Delgado Sancho, L., 2019. Technical proposals for selected new fertilising materials under the Fertilising Products Regulation (Regulation (EU) 2019/1009) - Process and quality criteria, and assessment of environmental and market impacts for precipitated phosphate salts & derivate. Sevilla.

Martinez-Sanchez, V., Kromann, M.A., Astrup, T.F., 2015. Life cycle costing of waste management systems: Overview, calculation principles and case studies. Waste Manag. 36, 343–355. doi:10.1016/j.wasman.2014.10.033.

Puig-ventosa, I., Freire-gonzález, J., Jofra-sora, M., 2013. Determining factors for the presence of impurities in selectively collected biowaste. doi:10.1177/0734242X13482030.

Riber, C., Petersen, C., Christensen, T.H., 2009. Chemical composition of material fractions in Danish household waste. Waste Manag. 29, 1251–1257.

Tampio, E., Marttinen, S., Rintala, J., 2016. Liquid fertilizer products from anaerobic digestion of food waste : mass , nutrient and energy balance of four digestate liquid treatment systems. J. Clean. Prod. 125, 22–32. doi:10.1016/j.jclepro.2016.03.127.

Tonini, D., Albizzati, P.F., Astrup, T.F., 2018. Environmental impacts of food waste: Learnings and challenges from a case study on UK. Waste Manag. doi:10.1016/j.wasman.2018.03.032.

Tonini, D., Dorini, G., Astrup, T.F., 2014. Bioenergy, material, and nutrients recovery from household waste: Advanced material, substance, energy, and cost flow analysis of a waste refinery process. Appl. Energy 121, 64–78. doi:http://dx.doi.org.globalproxy.cvt.dk/10.1016/j.apenergy.2014.01.058.

Tonini, D., Martinez-Sanchez, V., Astrup, T.F., 2013. Material resources, energy, and nutrient recovery from waste: are waste refineries the solution for the future?, Environmental Science and Technology. doi:10.1021/es400998y.
