# Supplementary material for: Quantitative sustainability assessment of household food waste management in the Amsterdam Metropolitan Area
Source: Resour Conserv Recycl. 2020 Sep;160:104854. doi: 10.1016/j.resconrec.2020.104854 (PMC7323620; doi:10.1016/j.resconrec.2020.104854)
Supplement: Supplementary file 2 [file mmc2.docx]

**Appendix B**

**Supplementary Methods**

**Quantitative sustainability assessment of household food waste management in the Amsterdam Metropolitan Area**

Davide Tonini^1^*, Alex Wandl^2^, Kozmo Meister^2^, Pablo Muñoz Unceta^2^, Sue Ellen Taelman^3^, David Sanjuan-Delmas^3^, Jo Dewulf^3^, Dries Huygens^1^

^1^European Commission, Joint Research Centre (JRC), Seville, Spain

^2^Technical University of Delft, Delft, the Netherlands

^3^Ghent University, Faculty of Bioscience Engineering, Department of Green Chemistry and Technology, Sustainable Systems Engineering Group (STEN), Ghent, Belgium

*Corresponding author: davide.tonini@ec.europa.eu

This Supporting Information document contains additional method insights.

For all the related Tables the reader is referred to the accompanying Excel-file "Appendix C – Supplementary datasets".

[Sustainability framework: list of impact categories & indicators 2](#_Toc27730588)

[System boundary example 2](#_Toc27730589)

[Method to model costs 3](#_Toc27730590)

[Method to model public acceptance & example 5](#_Toc27730591)

[Method to model accessibility of waste management system & example 7](#_Toc27730592)

[Method to model stakeholder participation & example 19](#_Toc27730593)

[Method to model disamenities & example 20](#_Toc27730594)

[References 26](#_Toc27730595)

## Sustainability framework: list of impact categories & indicators

| **Supplementary information available** | |
| --- | --- |
| Table C.1 | Main mass and energy flows in the scenarios assessed, expressed per functional unit (food waste generated in the Focus Area during a year). |
| **Explanatory notes** |  |

Note that the flow of C in organic fertiliser does not consider the subsequent mineralization following use-on-land. ww: wet weight. Electricity output and associated credit is reported as gross value. Values are rounded.

## System boundary example

| **Supplementary information available** | |
| --- | --- |
| Figure B.1 | System boundary exemplified for the case of scenario *cAD-PP* (centralised anaerobic digestion and post-processing for recovery of ammonium sulphate and biochar). Induced flows are indicated with continuous black lines, while substituted flows with dotted grey lines. |
| **Explanatory notes** |  |
| Figure B.1 illustrates the system boundary for the case of scenario *cAD-PP*. While the system boundary is in principle similar for the remaining scenarios, Table C.1 details the main mass, energy flows and conventional market products displaced with the outputs generated by managing the food waste generated annually in the Focus Area.    Figure B.1: System boundary exemplified for the case of scenario *CAD-PP* (centralised anaerobic digestion and post-processing for recovery of ammonium sulphate and biochar). Induced flows are indicated with continuous black lines, while substituted flows with dotted grey lines. | |

## Method to model costs

| **Supplementary information available** | |
| --- | --- |
| Equations B.1-B.8 | **-** |
| **Explanatory notes** |  |
| Following calculation principles for life cycle costing (Martinez-Sanchez et al., 2015), one-off budget costs (capital expenditure, i.e. CAPEX) are allocated equally between all tonnages of input-feedstock treated by a specific technology/process during the economic lifetime. This allows for obtaining a cost per unit of input-feedstock processed. This implies first calculating the annuity where the present one-off cost is annualised (*A*; Equation B.1) and then dividing the annuity by the annual usage rate of the technology to obtain a fixed cost per technology involved in the processing (*CI_f_*; Equation B.2). Fixed annual costs such as insurance and maintenance are also divided by the annual usage rate to obtain a cost per unit of input-feedstock treated. The variable budget costs are calculated by multiplying the amount of an item used in the technology (see technology inventory) by the corresponding unit-cost (see Table 15) to obtain a variable cost per item involved in the processing (*CI_v_*; Equation B.3). Associated transfer costs are calculated similarly (*CTI_v_*; Equation B.4). OELEX were calculated relying on the approach provided in Homes and Communities Agencies (2015) and using the average cost figures suggested for industrial areas (Equation B.5-to-C.7). These were Such OELEX represents a future cost that needs to be amortised and annualised to obtain a value consistently expressed per tonne of waste treated (Martinez-Sanchez et al., 2015). This implies using Equation B.8 where the one-off future cost *F (*i.e. *OELEX*) is annualised and then dividing this annuity by the annual usage rate *AUR* of the technology to obtain a fixed cost per tonne processed (*CI_f_*; Equation B.2). | |

$$A=\frac{P}{\left[ \frac{{(1+ir)}^{n}-1}{{ir(1+ir)}^{n}} \right]} \left[ \frac{€}{a} \right]$$

Equation B.1

$${CI}_{f}=\frac{A}{AUR} \left[ \frac{€}{t input} \right]$$

Equation B.2

$${CI}_{v}=I\cdot UPI \left[ \frac{€}{t input} \right]$$

Equation B.3

$${CTI}_{v}=I\cdot UTI \left[ \frac{€}{t input} \right]$$

Equation B.4

*AUR:* annual usage rate (t a^-1^)

*P*: present cost (€)

*n*: lifetime (a)

*ir*: interest rate (here assumed 5% conforming to ref.)

*I*: quantity of an item used in a technology per unit of input-feedstock treated (e.g. kg t^-1^)

*UPI*: unit-price of the item *I* (e.g. € kg^-1^).

*UTI*: unit-transfer for the item *I* (e.g. € kg^-1^).

$$OELEX=Removal of redundant services+Site clearance+Demolitions+Site investigations+Fees \left[ € \right]$$

Equation B.5

$$Site clearance=land occupation \cdot{UPI}_{clearance} \left[ € \right]$$

Equation B.6

$$Demolitions=land occupation \left( buildings \right) \cdot{UPI}_{demolition} \left[ € \right]$$

Equation B.7

*Fees*: 380,000 € (average of 90,000 - 670,000).

*Land occupation*: land occupied by site (m^2^)

*Land occupation (buildings)*: land occupied by buildings (m^2^)

*Removal of redundant services*: 90,000 € (average of 20,000 - 160,000)

*Site investigations*: 135,000 € (average of 10,000 - 260,000).

*UPI_clearence_*: unit-price of clearance (40 € m^-2^; average of 5 - 75).

*UPI_demolition_*: unit-price of demolitions (37,000 € m^-2^: average of 11,000 - 63,000).

$$A=\frac{OELEX}{\left[ \frac{{(1+ir)}^{n}-1}{ir} \right]} \left[ \frac{€}{a} \right]$$

Equation B.8

## Method to model public acceptance & example

| **Supplementary information available** | |
| --- | --- |
| Eqs. B.9-to-B.11 | **-** |
| Table C.6 | Spatial data used to model the collection of food and mixed waste in the reference scenario (*REF*). |
| Table C.7 | Spatial data used to model the collection of food and mixed waste in the scenarios I-to-V (*hCP*, *cCP*, *cAD*, *cAD-PP*, *MBT*). |
| **Explanatory notes** |  |
| Public acceptance is calculated in accordance with Equation B.9 knowing the annual fee paid for waste treatment by the households in a defined subarea (as € a^-1^; this information may be found in Table C.6 for the reference scenario *REF* and Table C.7 for the remaining scenarios).  $Public Acceptance=\frac{\sum_{i=1}^{n} {Cs}_{i}}{\sum_{i=1}^{n} {Cu}_{i}} x \frac{\sum_{i=1}^{n} {Xs}_{i}}{\sum_{i=1}^{n} ({Xu}_{i} x {Wsu}_{i})} x 100\% \left[ \% \right]$  Equation B.9  *i* – index of spatial subunit *n* – total number of spatial subunits in the focus area *Cs_i*: total yearly fee for the key waste flow per kg in spatial subunit *i* [€ kg^-1^]; *Cu_i*: total yearly fee for mixed waste per kg in spatial subunit *i* [€ kg^-1^]; *Wsu_i*: the weighted fraction of key flow (unsorted) in mixed waste in spatial subunit *i* [kg kg^-1^]; *Xs_i*: total weight of the key waste flow sorted (average) per actor in spatial subunit *i* [kg a^-1^]; *Xu_i*: total weight of mixed waste (yearly average) per actor in spatial subunit *i* [kg a^-1^].  For example, for scenario *cAD-PP*, 100% of the households in the Focus Area are served by a separate collection system but only a share of this by a door-to-door system (*DD*). Following the assumptions that food waste separately collected with door-to-door food is not charged and the overall fee per t of MSW remains the same as today's (*Fee_MSW,REF_*= 285 € a^-1^), the average *Public Acceptance* calculated for the whole Focus Area would then equal (assuming the share of food waste *W_su_* remains the same, i.e. 23%):  ${Fee}_{FW}=\left[ (DD\cdot0)+(100\%-DD) \right]\cdot{Fee}_{MSW, REF} \left[ \frac{€}{a} \right]$  Equation B.10  ${Fee}_{MSW}=\frac{{Fee}_{MSW}-{Fee}_{FW}\cdot w_{su}}{(100\%-w_{su})} \left[ \frac{€}{a} \right]$  Equation B.11  As an example, for the subunit Meerlanden the calculation would equal:  ${Fee}_{FW}=\left[ (67.4\%\cdot0)+(100\%-67.4\%) \right]\cdot284=92 \left[ \frac{€}{a} \right]$  ${Fee}_{MSW}=\frac{284-92\cdot23\%}{100\%-23\%}=341 \left[ \frac{€}{a} \right]$  $Public Acceptance=\frac{92}{341}\cdot\frac{13,839}{\left( 100,281\cdot23\% \right)}=16.3 \left[ \% \right]$  The results for the remaining sub-units and the whole Focus Area are reported herein:   \| **Parameter** \| **Unit** \| **AEB** \| **MEE** \| **MID** \| **PUR** \| **IND** \| **ORG** \| **AMA** \| \| --- \| --- \| --- \| --- \| --- \| --- \| --- \| --- \| --- \| \| *DD* \| % \| 0 \| 67.4 \| 64.7 \| 66.7 \| 66.7 \| 66.7 \| 42.2 \| \| *Fee_MSW,REF_* \| € a^-1^ \| 248 \| 284 \| 239 \| 215 \| 302 \| 101 \| 285 \| \| *Fee_FW_* \| € a^-1^ \| 248 \| 92 \| 84 \| 71 \| 100 \| 34 \| 165 \| \| *Fee_MSW_* \| € a^-1^ \| 248 \| 341 \| 285 \| 258 \| 362 \| 121 \| 321 \| \| Food waste separated (*X_s_*) \| t a^-1^ \| 33,433 \| 13,839 \| 19,414 \| 5,342 \| 9,475 \| 10,490 \| 91,992 \| \| MSW generated (*X_u_*) \| t a^-1^ \| 242,266 \| 100,281 \| 140,679 \| 38,707 \| 68,659 \| 76,018 \| 666,609 \| \| % Food Waste (*W_su_*) \| % \| 23% \| 23% \| 23% \| 23% \| 23% \| 23% \| 23% \| \| **Public Acceptance** \| **%** \| **60%** \| **16.3%** \| **17.7%** \| **16.6%** \| **16.6%** \| **16.6%** \| **30.8%** \| | |

## Method to model accessibility of waste management system & example

| **Supplementary information available** | |
| --- | --- |
| Figure B.2 | Example of a selected area of AMA with the spatial data collected for the accessibility calculation. AMA: Amsterdam Metropolitan Area. |
| Figure B.3 | Example of service area network analysis in a selected area of AMA. AMA: Amsterdam Metropolitan Area. |
| Figure B.4 | The location for food waste containers in the canals in Amsterdam city centre according to the proposed waste collection system. |
| Figure B.5 | Service area analysis in a selected area of AMA for distance ranges according to the proposed waste collection system. |
| Table C.17 | Spatial data used to model the collection of food and mixed waste in the *status quo* scenario (*REF*) and the accessibility to the waste management system. AMA: Amsterdam Metropolitan Area; NSC-FW: non separately collected food waste; SC-FW: separately collected food waste. |
| Table C.18 | Households and SMEs by distance range: *status quo*. AMA: Amsterdam Metropolitan Area. |
| Table C.19 | Accessibility of the waste management system: *status quo*. AMA: Amsterdam Metropolitan Area. |
| Table C.20 | Calculation of households per container and collection point for the proposed waste collection system. AMA: Amsterdam Metropolitan Area. |
| Table C.21 | Households by distance range: alternative scenarios. AMA: Amsterdam Metropolitan Area. |
| Table C.22 | Accessibility of food waste management system: alternative scenarios. AMA: Amsterdam Metropolitan Area. |
| Eqs. B.12-to-B.16 | - |
| **Explanatory notes** |  |
| The accessibility of the waste management system is measured by the distance from waste generation (households and SMEs) to the waste collection system (containers). It is expressed as the percentage of households or companies that need to cross defined distances to the collection points. The distance ranges are: 0-50, 50-100, 100-200, 200-300,.. , 700-800, and more than 800 metres. For the calculation of this indicator, the following assumptions are made:   - Households are differentiated into households with access to a garden and without a garden. If a household has access to a garden, it is assumed it uses a private above-ground roll container. In the AMA, this waste collection is done door-to-door. In these types of areas, it is assumed that there is, on average, one pick-up point every four houses (two on each side of the road). All these households will fall in the 0-50m range. - If a household does not have access to a garden, it is assumed to use public underground containers for waste disposal. This type of waste collection is considered curbside collection. Every underground container is a pick-up point for the waste collection truck. The distances from household to underground containers were calculated using geographic information systems (GIS) software. - Companies are differentiated into big companies and small and medium enterprises (SMEs). It is assumed that, due to their amount of waste, big companies use their own containers and collection is performed independently. Therefore, they are not included in the calculation. The behaviour of SMEs will be considered the same as private households, although it is assumed that they use the public underground containers to dispose of their waste since municipalities do not provide companies with private roll-containers.   ***Preparation and availability of data***  Four sets of data are used for the spatial calculations of the accessibility indicator:   1. Location of the collection points 2. Walkable street network 3. Address points of the households 4. SMEs’ locations   (1) For the location of the collection points, spatial data was requested from all waste operators in all wastesheds in the AMA. However, only the data for some neighbourhoods could be collected. The spatial dataset includes 9,497 collection points for residual waste (in 30 neighbourhoods) and 188 collection points for food waste (in 9 neighbourhoods).  (2) The walkable street network is a dataset with all roads and streets in the AMA, excluding highways. ArcMap was used to create the network dataset.  (3) As mentioned previously, households are divided into households with or without access to a garden. Spatial data is obtained from the basic registration for addresses and buildings (BAG, in Dutch *Basisregistratie Adressen en Gebouwen*). Addresses (point data set ‘BAG adres’) are used to determine the number of households. The Urban Mining Model pre-processed data is used (Geldermans et al., 2019) to classify the buildings according to building type. The following steps were performed:  *Spatial operations for the selection of households:*     1. The initial number of buildings according to the pre-processed data of the urban mining model is 690,394. Polygons (buildings) have a function assigned (office, residential, industry, etc.) according to BAG data base. The urban mining model includes a classification on the building type (office building, apartment, row-house, semi-detached house, single-family house, other uses). 2. Only residential buildings are considered. ‘Select by attributes’ function is used in ArcMap to select attribute class = apartment OR row-house OR semi-detached house OR single-family house. 3. The resulted dataset of polygons (buildings) is joined spatially with households (point data set ‘BAG adres’) using ‘spatial join’ ‘one-to-one’ function in ArcMap. The result is 593,723 buildings with 1,066,955 households. The number of households (total count) per polygon is kept in a column of the attribute table. 4. The number of households is classified in two groups: those with or without access to a garden. Apartment buildings and dwellings in upper floors of row-houses are considered not to have access to a garden. These are selected using ‘select by attributes’ function in ArcMap (class = ‘apartment’ OR class = ‘row-house’). A new attribute is created “HH-no-garden” and a function with the previous assumption is applied to calculate the number of households. Total number of households without garden is 508,858. 5. Only households in the neighbourhoods with spatial data on collection points are selected. This is done using ‘Select by location’ function in ArcMap using as ‘target layer’ the building polygons and as ‘source layer’ the neighbourhoods where we have spatial data on collection points. It results in 326,064 households for areas with information on residual waste collection, and 14,189 households in areas with information on food waste collection. 6. The final polygons (buildings) with the households’ information are transformed into two datasets of points. One with the households in the neighbourhoods with information on food waste collection (14,189 points), another with the households in the neighbourhoods with information on residual waste collection (326,064 points).   (4) As mentioned earlier, the waste collection for companies depends on their size. For their locations, the bvdinfo data from the Orbis company database is downloaded and georeferenced into a point feature dataset. The database classifies companies according to their size. SMEs are selected with ‘select by attribute’ function in ArcMap. SMEs in the neighbourhoods where we have spatial data on collection points are selected. This selection results in 3,115 SMEs in areas with food waste collection data and 64,664 SMEs in areas with residual waste collection data. See Table C.17 for detailed information of all the results of these spatial operations classified by wasteshed. See Figure B.2 for a visual example on the type of data available in the AMA case that was prepared for the calculation of the indicator.  ***Calculation method***  A ‘service area network’ spatial analysis is run on ArcMap. The collection points are used as ‘origins’ and the walkable street network as ‘network data set’. The service area is defined according to the distance ranges defined earlier. Two different operations are run; one for the food waste collection system and another for residual waste collection system. The results of the analyses are two sets of isochrones by distance ranges according to the network dataset. These are exported as polygons. See Figure B.3 for a visual example of the spatial operation.  The polygon dataset is used to calculate the number of households and SMEs per distance range. The spatial calculation counts how many location points fall inside each polygon, which represents a distance range, according to the service area network. A spatial function ‘select by location’ is run, using the polygon for each distance range as ‘source layer’ and the households/SMEs points as ‘target layer’. The calculation is run only in the neighbourhoods where spatial data on collection points was found. The results of the areas with spatial data are classified by distance ranges (i). The percentages of households per distance range are calculated per wasteshed (Equation B 12). The total percentages for the AMA are calculated as a sum of the areas where spatial data was found.  ${R^{*}(\%)}_{i}={{HH}^{*}'}_{i}\div{{HH}^{*}}_{tot}$  Equation B.12  *R^*^(%)_i_*: percentage of households+SMEs per distance range (i) in areas with spatial data in the wasteshed  *HH^*^’_i_*: number of households without garden and SMEs in a distance range (i) in areas with spatial data in the wasteshed  *HH^*^_tot_*: total number of households and SMEs in areas with spatial data in the wasteshed (including households with garden and without garden)  These results, calculated separately for the food waste collection system and the residual waste collection system, consider the total amount of SMEs and households without access to a garden. As mentioned earlier, households with access to a garden are included in range 0-50m (door-to-door collection). In order to add them to the previous spatial calculation, the results are extrapolated to the total number of households in each wasteshed. For the range 0-50m, Equation B.13 is used. For the remaining ranges, Equation B.14 is used.  ${R(\%)}_{0-50}=\left[ \left( {HH'}_{tot}\times{R'(\%)}_{0-50} \right)+{HH''}_{tot} \right]\div{HH}_{tot}$  Equation B.13  ${R(\%)}_{i}=\left( {HH'}_{tot}\times{R'(\%)}_{i} \right)\div{HH}_{tot}$  Equation B.14  *R(%)_i_*: percentage of households per distance range (*i*) in the wasteshed  *R’(%)_i_*: percentage of households per distance range (*i*) in areas with spatial data in the wasteshed  *HH’_tot_*: total number of households without garden and SMEs in the wasteshed  *HH’’_tot_*: total number of households with garden in the wasteshed  *HH_tot_*: total number of households and SMEs in the wasteshed (including households with garden and without garden)  SMEs behaviour is assumed the same as that of the households without garden.  Table C.18 classifies all households and SMEs according to distance ranges and wastesheds. The wastesheds for which no data was collected are left blank.  Besides all previous assumptions, it is also assumed that there is no separate collection of food waste in AEB wasteshed. Therefore, in AEB all SMEs and households fall into the highest distance range (>800m).  **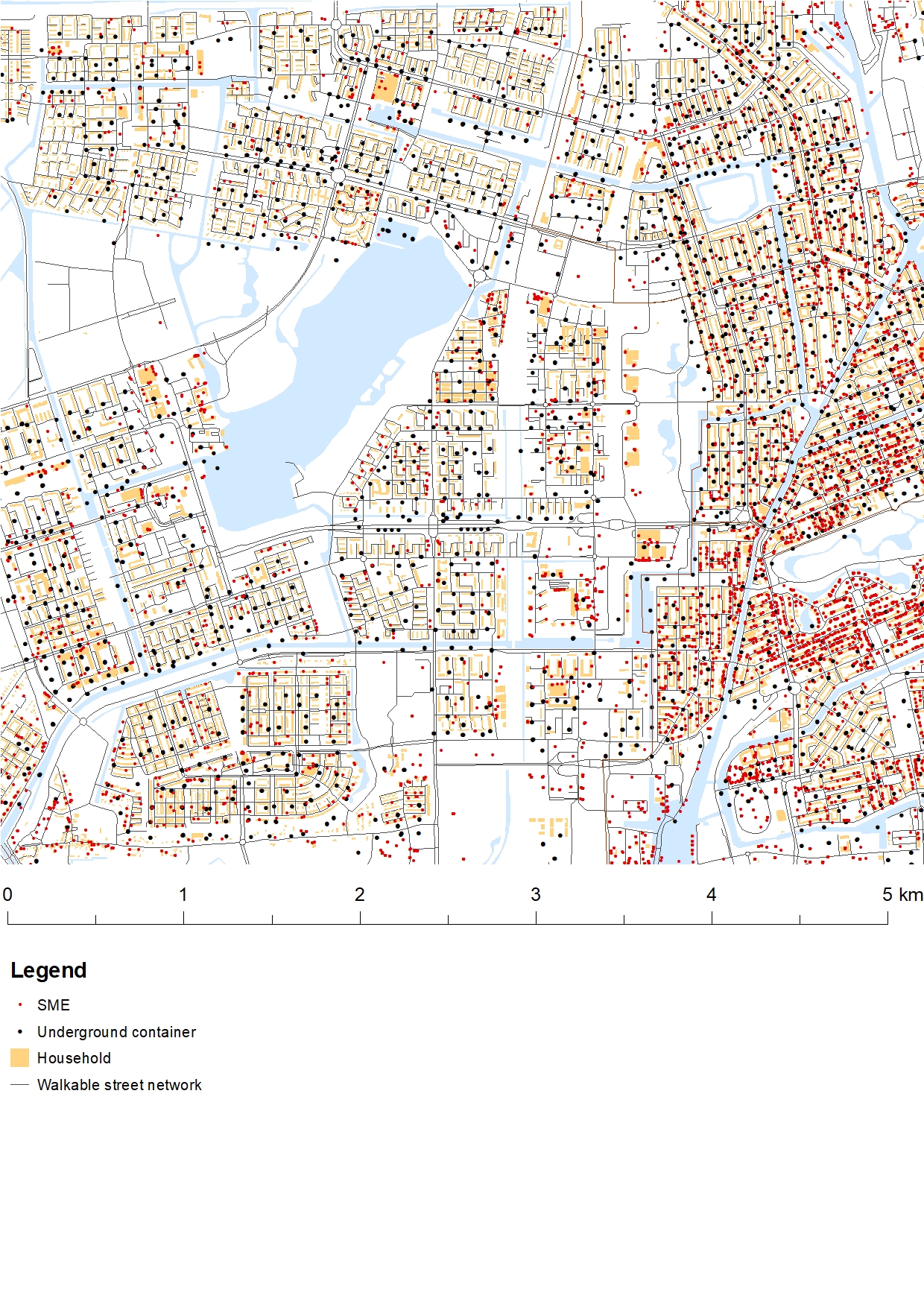Figure B.2: Example of an area in AMA with the spatial data collected for the accessibility calculation. The data showed in the map corresponds to residual waste containers (underground containers) as a points data set, households (without a garden) as polygons (they were later transformed into points), SMEs as points and the walkable street network as polylines. AMA: Amsterdam Metropolitan Area.**  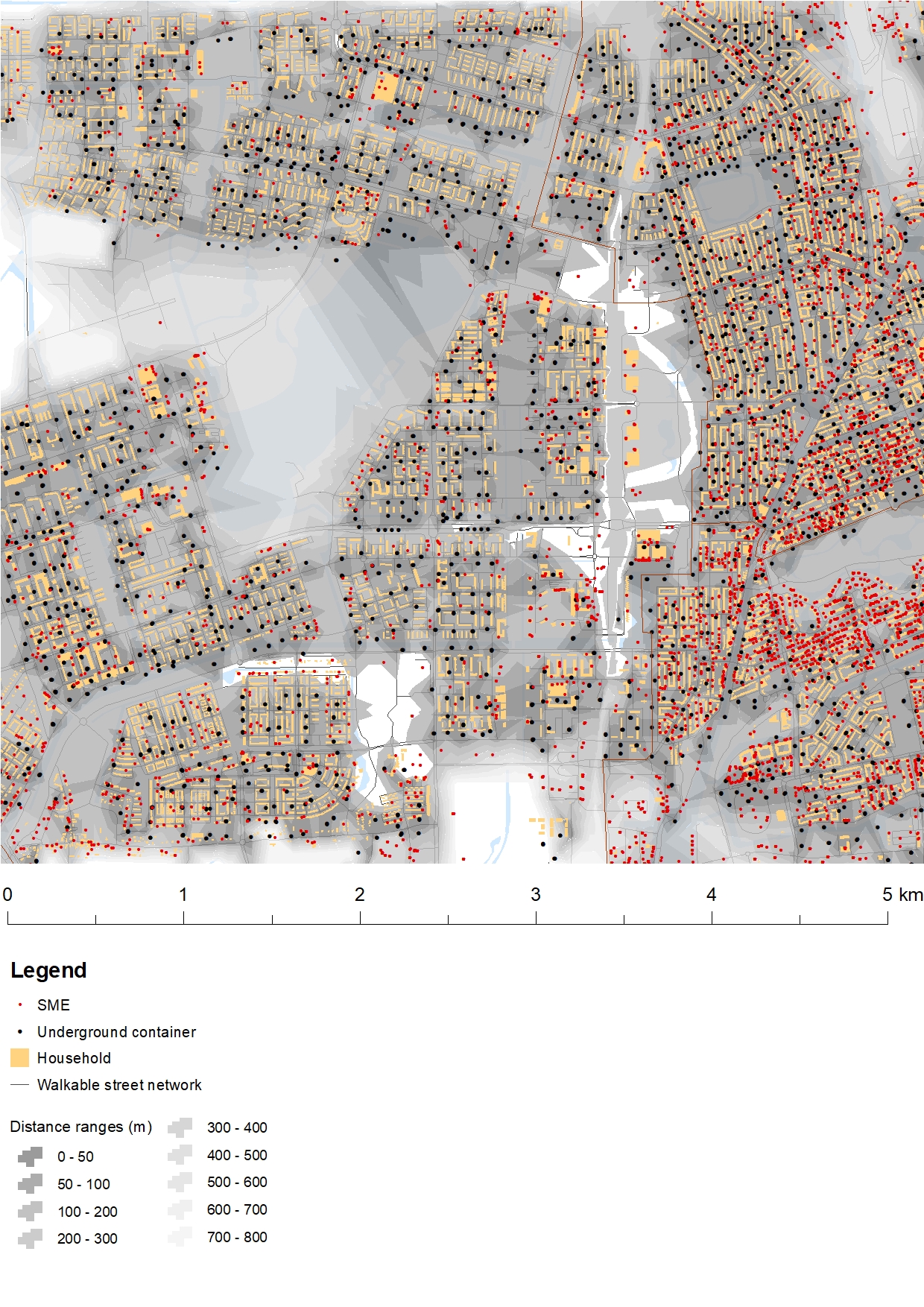  Figure B.3: Example of service area network analysis in an area in AMA. The data showed in the map corresponds to the same layers as Supplementary Figure B.2 plus the isochrones polygons for each distance range, according to methodology. This last data set is transformed later into isolated polygons to select households and SMEs within each range. AMA: Amsterdam Metropolitan Area.    ***Other information derived with the spatial information***  Using the information described above we also derived *Number of households per collection point* and *number of households per container*. As mentioned earlier, the following assumptions are considered:   - Each household with access to a garden has an above-ground roll container. - For households with access to a garden, there are 4 households per pick-up point (door-to-door collection). - For households without access to a garden, there is one pick-up point for every underground container (curbside). - In the AEB wasteshed there is no separate food waste collection. - SMEs have the same behaviour than households without access to a garden.   Each of the indicators is a weighted average of the number of households per container and per collection point. Two areas are considered: areas with households that have access to a garden and areas with households that don’t have access to a garden. For the latter, the calculation is performed only in the wastesheds with spatial data. It is assumed that the remaining wastesheds have similar characteristics. For wastesheds where no spatial data are available, an average on the other wastesheds is used. Households per collection point (*HH_cp_*) are calculated according to Equation B.15:  ${HH}_{cp}=\%HH^{'}\times\left( {HH'}^{*}\div{CP'}^{*} \right)+\%HH''\times\left( {HH''}_{tot}\div{CP''}_{tot} \right)$  Equation B.15  *HH’’*: households with access to a garden  *HH’*: households without access to a garden and SMEs  *HH’^*^*: households without access to a garden and SMEs in areas with spatial data  *CP’^*^*: number of collection points (underground containers) in areas with spatial data  *CP’’*: number of collection points for households with access to a garden (roll-containers)  Equation B.15 is also applied to calculate households per container, using the number of containers instead of the number of collection points. Table C.17 reports results for the *status quo*, classified by wasteshed and type of waste.  Both figures are higher for food waste than residual waste due to the fewer food waste containers compared to residual waste containers. The AEB figures for residual waste (there is no separate food waste collection) are very high due to the presence of dense urban areas (Amsterdam city centre) and, therefore, high household density. Moreover, according to the assumptions for these areas, curb-side collection is applied to apartment buildings, which share collection points (or containers) with other buildings. Indicators, therefore, vary depending on the type of space in each wasteshed. The most urbanized wastesheds, such as AEB and Middenmeer, have higher values for both indicators, whereas suburban low-density wastesheds, such as Orgaworld or Meerlanden, show lower indicators. Households in suburban areas have their own containers and the amount of pick up points is also higher than in urban areas.  ***Accessibility of waste management system: status quo***  The accessibility (*A*) to waste management system indicator is calculated conforming to Taelman et al. (2019), Equation B.16.  $\text{A(\%)}=\sum_{i=1}^{10} {wa}_{i}\times X_{i}$  Equation B.16  where *wa_i_* represents the ‘weights’ of the distance ranges *i* and *X_i_*, the % of distances door-to-collection points. Table C.19 reports the calculations for AMA case under this methodology as well as the resulted accessibility to the waste management system indicators. In the wastesheds where there is no data, an average of all the other wastesheds is applied. Final indicators are presented here, classified by type of waste (food waste / residual waste) and type of origin (households / companies). Accessibility to the waste management system is much lower for food waste than for residual waste due to the low density of underground containers.  ***Proposed waste collection system***  The aim of this scenario is to increase households’ and SMEs’ food waste collection by increasing the accessibility to the system. There are specific assumptions and measures considered for the proposed changes:   - The same assumptions as in the *status quo* apply to households without access to a garden: They use roll-containers and collection is performed door-to-door, with 4 households per pick-up point. - SMEs are considered to have the same behaviour as households without a garden.   In order to increase accessibility, three different measures are proposed in the new scenario:  (1) A new separate food waste collection system is proposed in the AEB wasteshed. In the centre of Amsterdam, a new food waste collection system, using floating containers, is proposed for households that do not have access to a garden and for SMEs. Food waste is collected by boat or truck from these points. Outside the centre of Amsterdam, accessibility to the food waste collection system is increased by decreasing the number of households per container and collection point to the level of current residual waste collection. This results in an increase in the number and density of waste containers.  (2) Door-to-door collection is implemented in high density areas in all the other wastesheds. Areas with more than 5,000 inhabitants per square km, which is a high density in the Netherlands, have door-to-door collection for both households with and without access to a garden. This means that every apartment building, row house, semi-detached house and single-family house have one container where all households and SMEs in the building will dispose their food waste. In these areas, SMEs and households without access to a garden have one collection point per building, whereas households with access to a garden have one collection point every four households.  (3) In the remaining wastesheds (other than AEB) where population density is lower than 5,000 inhabitants per square km, accessibility to food waste collection system is increased by decreasing the number of households per container and collection point to the level of residual waste collection.  Based on these assumptions, calculations are applied differently in three different areas.  (1) For the city centre of Amsterdam, a new collection system is proposed and spatially designed. A “service area network analysis” is used to design the collection system in the centre of Amsterdam. The origins are the new collection points, based on their proximity to bridges, and the network is the walkable street network. According to the Amsterdam Municipality, the maximum walkable distance to a container must be 150m (Raad van State, 2018; Coertenraede, 2018). The “service area” is set up to a maximum 150m and the containers are placed to provide access to households within the “service area,” see Figure B.4. The isochrones for the distance ranges are calculated using the previous methodology (see previous step *calculation method*). These isochrones are transformed into polygon features and used to calculate the number of households within (see Figure B.5). The number of households within each distance range is reported in Table C.21.  (2) High density areas are selected using CBS data. The ‘select by attributes’ function is used, “Inhabitants (*inwoners in Dutch*) > 5000”, in the neighbourhood scale (*wijk* in Dutch). This results in 33 neighbourhoods with 121,149 households. These neighbourhoods are located in three different wastesheds (Middenmeer, Purmerend and Indaver). Spatial calculations on the number of households with and without access to a garden as well as number of buildings and SMEs per wasteshed are performed. Results are reported in Table C.20.  (3) Households in non-dense areas are calculated by subtracting the households in high density areas to the total number of households. This operation is also carried out for households with and without a garden, SMEs and buildings. Results are reported in Table C.20.  With all this new collected spatial data and previous calculations, the indicators on number of households per collection point and per container are recalculated independently in these four areas: AEB-Amsterdam-centre, AEB-outside-Amsterdam-centre, dense neighbourhoods and non-dense neighbourhoods. All values of different areas within the same wasteshed are weighted and reported in Table C.20.  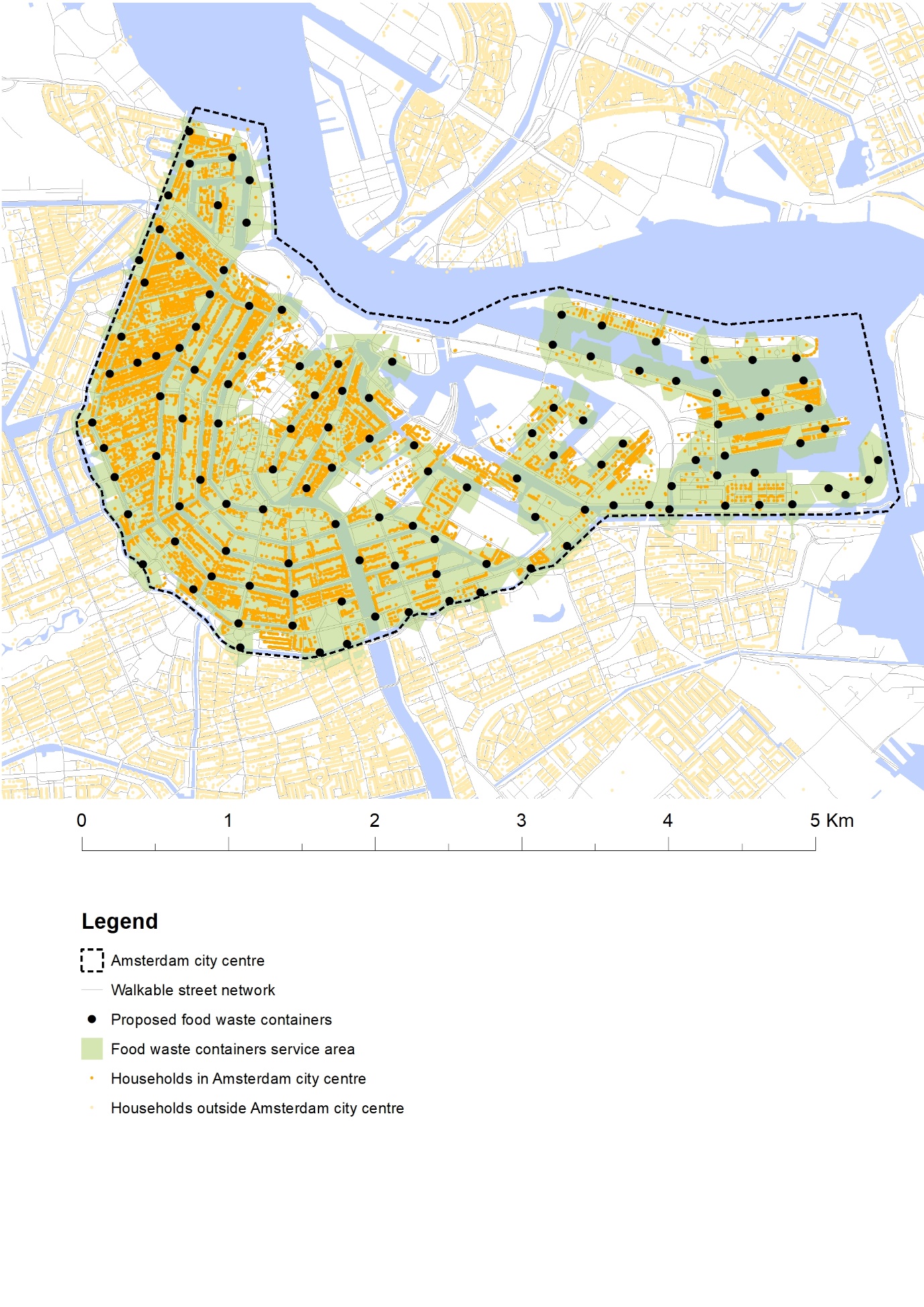  Figure B.4: Proposed location for food waste containers in the canals in Amsterdam city centre according to the proposed food waste collection system. They are located so that the minimum service area (150m) serves most households within the city centre. The gaps are areas where there is not canal close enough, there are not buildings, or there are buildings without households (industries or other functions that do not use the municipal waste collection system). Black dots are the proposed containers and orange points are households. AMA: Amsterdam Metropolitan Area.  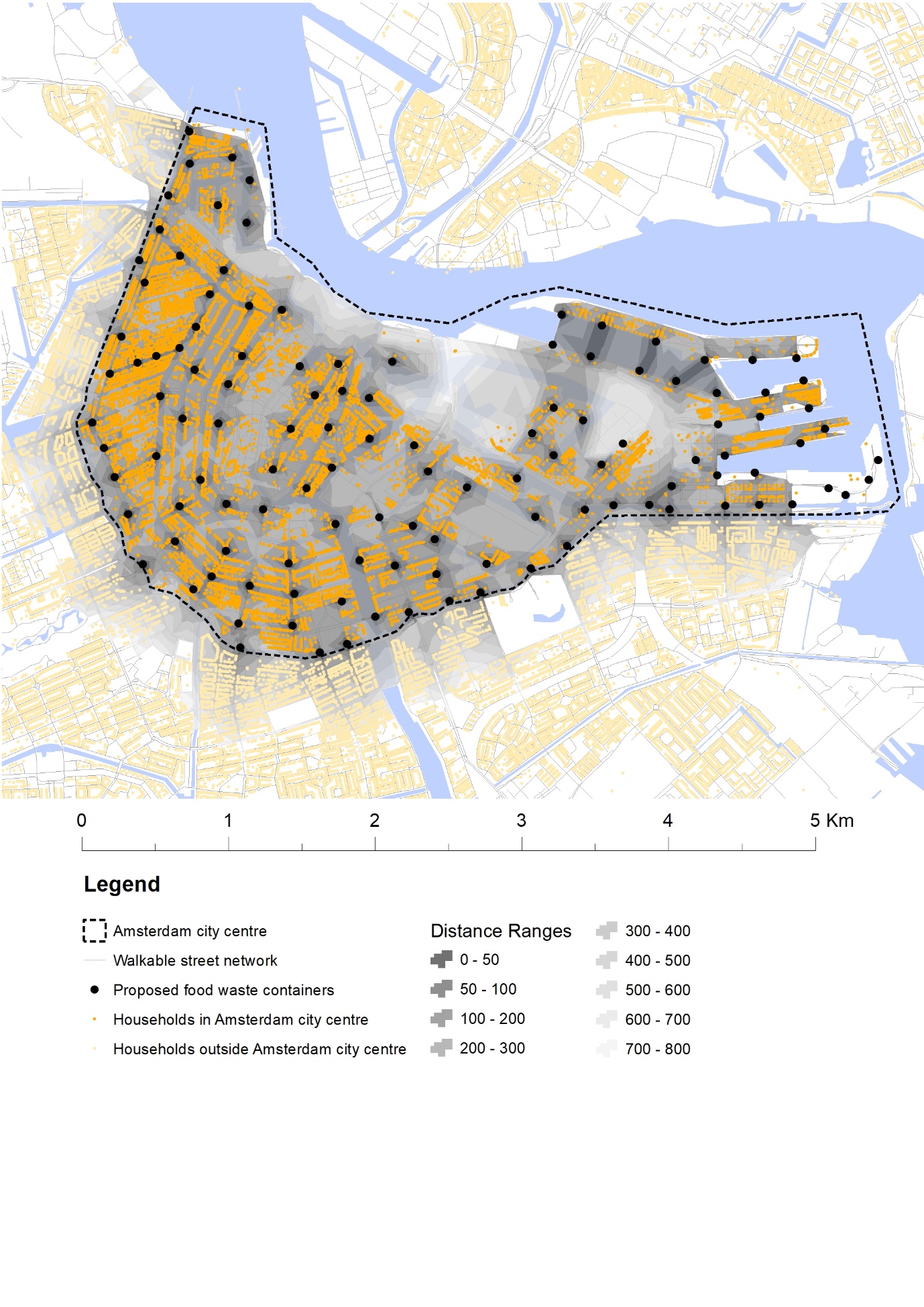  Figure B.5: Service area analysis in an area in AMA for distance ranges according to the proposed food waste collection system. This is used to calculate accessibility to the waste management system. The data showed in the map corresponds to the same layers as Supplementary Figure B.4, but the service area is transformed into isochrones that go beyond 150m. AMA: Amsterdam Metropolitan Area.  Under the proposed scenario, the wastesheds undergo a considerable decrease in the number of households per collection point or container, which have an impact on the shared costs of the system. The AEB wasteshed still has high indicator values due to the high density of Amsterdam city centre, the limitations of waste collection due to the spatial characteristics (canals) and the presence of many other dense areas in the rest of the wasteshed.  The data are also used to calculate the number of households and SMEs that fall in each range of accessibility. The values calculated in the new ‘service area network analysis’ are considered for AEB-Amsterdam-centre. In dense neighbourhoods, all households now fall into the range 0-50m because collection is performed door-to-door. In non-dense neighbourhoods and AEB-outside-Amsterdam-centre, the SMEs and households without access to a garden are sorted according to the percentages of residual waste collection system accessibility in the *status quo* (Table C.19) and include all households with access to a garden in range 0-50m (Table C.21). Accessibility indicators are calculated using the same methodology as for the *status quo* and reported in Table C.22. Under these assumptions, the accessibility indicator increases compared to the *status quo*. | |

## Method to model stakeholder participation & example

| **Supplementary information available** | |
| --- | --- |
| Table C.26  Eqs. B.17-B.18 | a) Calculation of stakeholder participation and b) aggregation per sector. SP: Stakeholder Participation.  - |
| **Explanatory notes** |  |
| This indicator assesses the rate of stakeholders participation (*SP*) in the development of eco-innovative solution per specific and aggregated actor groups. It is calculated as the share of stakeholders that participated in a workshop compared to the total number of invited stakeholders and is calculated according to Equation B.17:  $SP=S_{a}/S_{i}$  Equation B.17  S_a_: number of stakeholders that attended a workshop.  S_i_: number of stakeholders that were invited to a workshop.  If multiple workshops (*w*) have to be considered, Equation B.18 is to be used:  ${SP}_{t}={\frac{\sum_{w=1}^{n} ({S_{a1}}/{S_{i1})+ ({S_{a2}}/{S_{i2})+\ldots+ ({S_{awn}}/{S_{iwn})}}}}{n}}$  Equation B.18  *SP_t_*_:_ the total stakeholder participation rate  *w*: number of workshops to be considered.  *S_a_*: number of stakeholders that attended a workshop.  *S_i_*: number of stakeholders that were invited to a workshop.  In the case of Amsterdam, two workshops (*W_2_* and *W_3_*) have been included in the calculations, as those workshops focussed on the development of eco-innovative solutions. Table C.26a presents the numbers of invited and attending stakeholders according to different types of expertise as well as stakeholder participation for the individual workshops and across all workshops. In total, 110 stakeholders were invited, 33 attended workshop 2 and 21 attended workshop 3. The resulting participation rates are: 0,3 (*w_2_*), 0,19 (*w_3_*) and 0,25 for the whole process of EIS development. Table C.26b shows the same data aggregated for stakeholder groups. To achieve a high participation rate for the workshops, we invited sometime multiple potential participants from the same institution. In the calculation of the indicator, we counted this as one single invitation. The assumption is that for the *status quo,* *SP* is 0. If a scenario includes an eco-innovative solution discussed in the workshop the specific *SP* is assumed. | |

## Method to model disamenities & example

| **Supplementary information available** | |
| --- | --- |
| Figure B.6 | Example of spatial data used for the analysis of landscape disamenities in AEB (incinerator) + Greenmills (anaerobic digestion & composting) facilities. |
| Table C.23 | Landscape disamenities: Linear regression model - Correlation analysis. |
| Table C.24 | Landscape disamenities: Linear regression model - Excluded variables analysis. |
| Table C.25 | Landscape disamenities:Calculation of the property value loss nearby the waste treatment facilities. |
| Eqs. B.19-B.20 |  |
| **Explanatory notes** |  |
| This indicator quantifies the disamenities in monetary terms, evaluating the effects of waste management facilities on property prices using hedonic prices, which is an indirect quantification of the preference (Taelman et al., 2019). Five different ranges are defined to calculate disamenities: 0-1km, 1-2km, 2-3km, 3-4km, 4-5km. Waste treatment plants in the AMA are classified according to their type of process:   \| **Name** \| **Area (m2)** \| **Type** \| \| --- \| --- \| --- \| \| Greenmills \| 45,090 \| Composting \| \| HVC Purmerend \| 24,150 \| Composting \| \| Orgaworld \| 648,556 \| Composting \| \| Orgaworld \| 11,985 \| Anaerobic digestion \| \| HVC Alkmaar \| 165,380 \| Incinerator \| \| HVC Middenmeer \| 277,311 \| Anaerobic digestion + Composting \| \| AEB \| 102,207 \| Incinerator \| \| Meerlanden \| 77,694 \| Anaerobic digestion + Composting \| \| Indaver \| 31,095 \| Anaerobic digestion + Composting \| | |
| Four cases are selected:   - AEB (Incineration plant) + Greenmills (Composting plant). Due to their proximity, their buffer zones are combined - HVC Alkmaar: Incineration plant. - Meerlanden: Anaerobic digestion plant + Composting plant. - Purmerend: Composting plant.   Buffers are spatially calculated according to the location of the plants. Both residential and non-residential buildings fall inside these buffers. For each selected case, 20 residential buildings are selected within each distance range. Nevertheless, there are some cases in which there are fewer, or none, residential buildings within some range. This happens in closer ranges when the facility is located in an industrial or isolated area. The selection is made on a similar base between ranges, if possible. For example, a similar amount of row housings or apartment housings in each range. See Figure B.6 for an example of the spatial data used for this analysis in the case of the AEB incinerator + Greenmills composting facility.  The value loss (or increase) for each property could be influenced by several characteristics and not only by the proximity of a waste management facility (Rivas et al., 2017). Based on Rivas Casado et al. (2017), the model includes several variables. A database is created for the selected buildings in the buffer zones of each plant including the following variables:   - Building age (<https://www.wozwaardeloket.nl/index.jsp>) - Housing type (according to the Urban Mining Model developed in Geldermans et al., 2019) - Linear distance to plant (calculated using GIS) - Area (<https://www.wozwaardeloket.nl/index.jsp>) - Liveability (<https://www.leefbaarometer.nl/kaart/#kaart>) - Value of the property: WOZ2018 (<https://www.wozwaardeloket.nl/index.jsp>)   The database is divided into four datasets (one per waste management facility) and transformed into SPSS files. A linear regression is run using WOZ2018 value as a dependent variable and ‘Year of construction,’ ‘Liveability index’, ‘Type of dwelling’, ‘Linear distance’ and ‘Area’ as independent variables. For each case, a collinearity analysis is run (see Table C.23).  In the case of the incinerators, there is significant correlation at the 0.05 level in both cases. In the case of a composting facility there is no significant correlation between property value (WOZ 2018) and distance to the waste management facility (linear dist). To test the influence of the amount of data collected, two linear regressions are carried out for similar facilities. In the case of the incinerators, independent (one per facility) and combined (one for the two facilities together) linear regressions are carried out. In the case of the Composting facility, only a combined analysis of the regression is carried out.  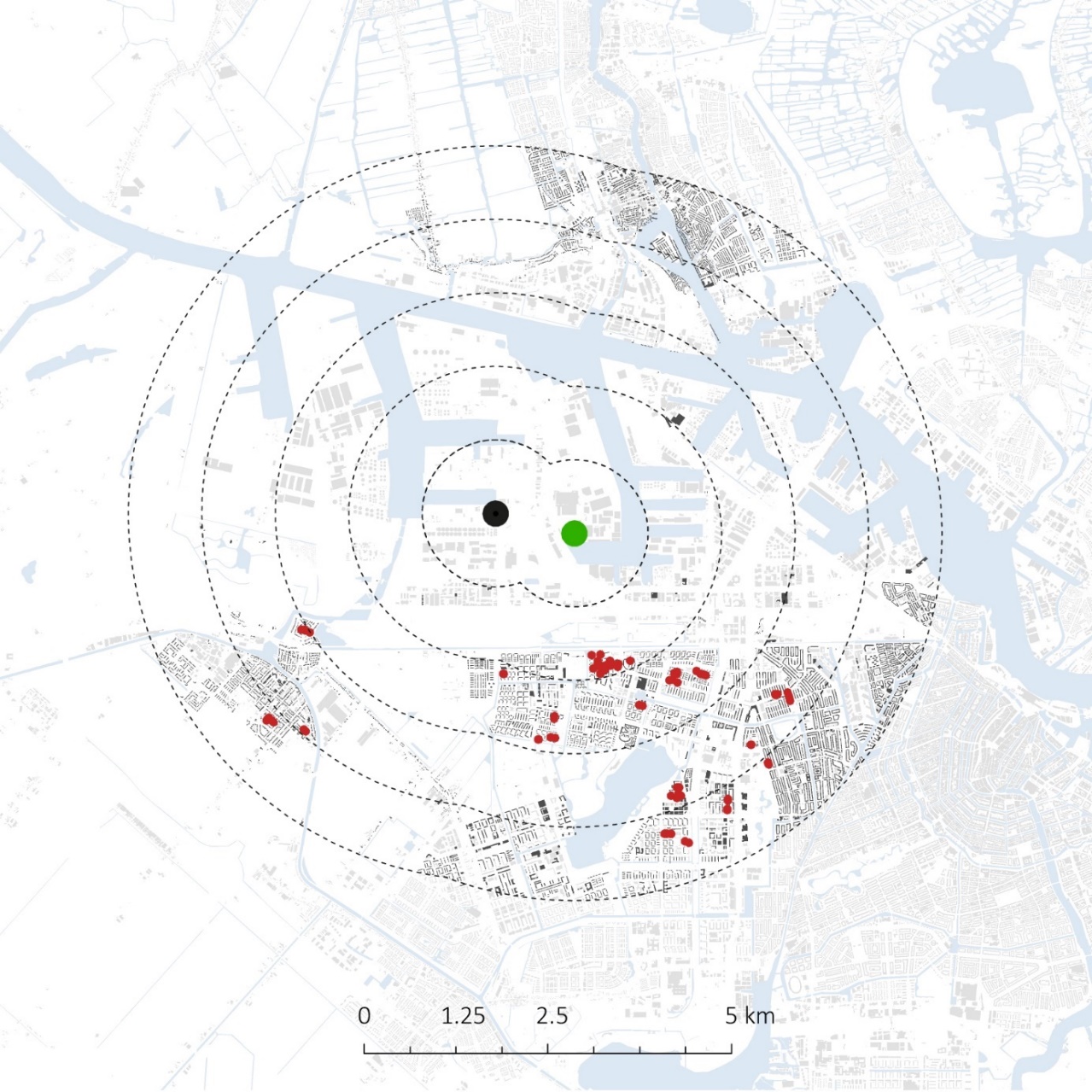  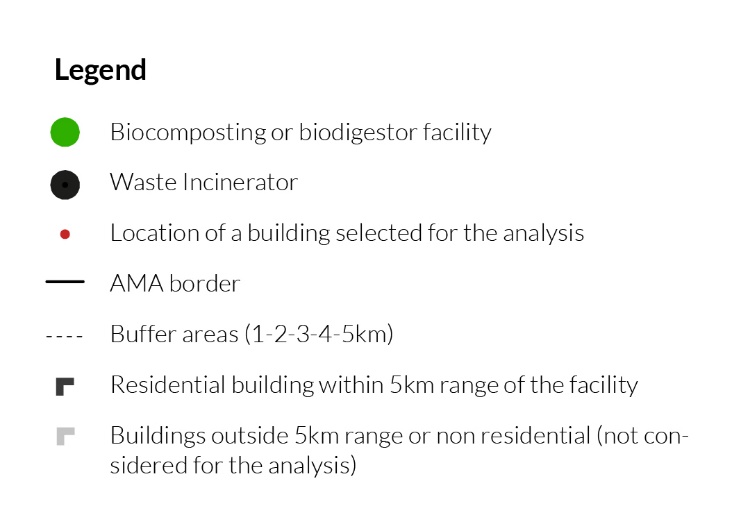  Figure B.6: Example of spatial data used for the analysis of landscape disamenities for the case of the area where the AEB (waste incinerator; black dot) and Greenmills (biocomposting and digestor; green dot) facilities are located.  ***Incinerators (independently)***  First, outliers are checked with ‘Mahalanobis Distances’ (Multivariate outliers). There are none found in any of these two facilities. Secondly, a ‘collinearity check’ is run in both cases. Despite the high values for HVC Alkmaar (see Table C.24), there is no collinearity. Two operations are carried out. First, in the case of Alkmaar, the ‘casewise diagnosis’ detects two outliers. Second, there are two variables (year of construction and liveability) that affect the results of the regression depending on the order in which the variables are introduced. Two calculations are run for each facility; one including all five variables, another without the two altering variables. The ‘unstandardized B coefficients’ (relation between the linear distance to waste management facility and the property value) for each of the linear regressions are as follows:  AEB:   - Linear regression with all 5 variables: **1€ every 26,16 m** - Linear regression with 3 variables: **1€ every 24,59m**   HVC Alkmaar:   - Linear regression with all 5 variables: **1€ every -6,46m** - Linear regression with 3 variables and no outliers: **1€ every 7,83m**   Whether the independent variables (liveability and year of construction) are included or not in the model for HVC Alkmaar considerably affects the linear regression.  ***Incinerators (combined data)***  As with the isolated incinerator, there is a significant correlation at the 0.01 level between value and distance to the facility (see Table C.23). The same steps are carried out as before; a multivariate outlier check and collinearity check, yielding in no observed collinearity. The same process for the linear regression is carried out. Outliers are identified by the ‘casewise diagnosis’ and removed from the model. There is one variable (‘year of construction’) that affects the results of the regression depending on the order in which the variables are introduced. Two regressions are calculated. One with ‘year of construction’ and another without it.The unstandardized B coefficients (relation between the linear distance to waste management facility and the property value) for each of the linear regression are as follows:   - Linear regression with all 5 variables: **1€ every 37,99m** - Linear regression with 4 variables and no outliers: **1€ every 36,43m**   Not only does the number and order of variables affect the results less when using combined data, the presence or absence of the outliers has a lighter influence in the regression in comparison to running it on isolated facilities. When dealing with the combined data, the model appears to be more robust.  ***Composting facilities (combined data)***  Even though the model, when applied to independent composting facilities, showed there was no significant correlation between property value and distance to the facility, the linear regression is applied to the combined data of the two composting facilities (Meerlanden and Purmerend), to see if, similar to the incinerators, the model works better when using a bigger dataset. First, correlation is checked. In contrast to the results when running the model on the composting facilities independently, there is a significant correlation between distance to the facility and the property value (see Table C.23). No collinearity is observed (see Table C.24). The same process as before is carried out to run the linear regression. Outliers are also identified by the ‘casewise diagnosis’ and removed from the model. There are two variables (‘year of construction’ and ‘liveability’) that affect the results of the regression depending on the order in which they are introduced. Two regressions are calculated, one with all three variables and another one with only three variables. The ‘unstandardized B coefficients’ (relation between the linear distance to waste management facility and the property value) for each of the linear regression are as follows:   - Linear regression with all 5 variables: **1€ every - 0,44m** - Linear regression with 3 variables and no outliers: **1€ every 1,12m**   Even if the model is more consistent than applied on independent facilities, the values are very high in comparison to incinerators, despite the smaller size of composting facilities. 1€ of value lost every 1,12m that a property gets closer to the facility, equals to a value decrease of 892.86€ per km. Combined incinerators show a value decrease of 27,46€ per km. This could be due to other variables not included in the model that affect property value in the case of the composting facilities, or the need of a bigger database to have more reliable coefficients. Nonetheless, no significant correlation between property value and distance to a composting facility is assumed.  ***Calculation of the landscape disamenities indicator***  The total value loss caused by the proximity to a waste management plant depends on the sum of the value losses of houses in each distance range. This, depends, at the same time, on the standardized coefficient calculated for each type of facility. Houses are selected according to the 1km ranges in order to calculate the total impact of the facility in their property value. The number of houses per distance range is spatially calculated with “select by location” function in ArcMap, using households in residential buildings according to BAG (*Basisregistratie Adressen en Gebouwen*) data base as “source layer” and the different buffers as “target layer”. The standardized coefficient depends on distance which is divided equally in five buffers. Therefore, the coefficient value must be distributed proportionally among five different ranges. The further away the range is to the facility, the lower the disamenities value. A direct relation between ranges’ coefficients is assumed: Range 5 (0-1km) coefficient is five times range 1 (4-5km) coefficient, range 4 (1-2km) is four times range 1 (4-5km), and so on. Because buffers are distributed equally in ranges of 1km, it is assumed C is an average of all *C_i_*. According to these two assumptions, in an area divided in five equal buffer zones (Equation B.19):  $Ci=C\times\frac{i}{3}$  Equation B.19  Where *C_i_* is the standardized coefficient according to the linear regression and *Ci* is the specific standardized coefficient for each distance range (*i*). Landscape disamenities indicator is calculated using Equation B.20, according to the specific standardized coefficients. It is assumed that beyond 5km there is no influence of the facility in the property value.  $Ld=\sum_{i=1}^{5} \left( {Nh}_{i}\times C_{i} \right)$  Equation B.20  *Ld*: Landscape disamenities value  *i*: Distance range  *Nh*_i_: Number of houses within each distance range  *C_i_*: Standardized coefficient defined for each distance range  The standardized coefficients calculated based on the ‘unstandardized B coefficients’, extrapolating the results to € per km. The final coefficients are:   - Incineration plant (based on Alkmaar): 127,83€ per km - Incineration plant + Anaerobic digestion (based on AEB): 41.05€ per km - Composting plants (it is assumed there is no correlation in these cases): 0€ per km   The total property value loss is then calculated for each plant using the specific standardized coefficients by type of facility (see Table C.25). These values are then divided by the total amount of waste treated by the facility over the entire life time (i.e. 20y) to obtain a money loss per tonne of food waste treated. | |

## References

Coertenraede, T., 2018. Rethinking waste. Technical University of Delft.

Geldermans, B., Wandl, A., Meister, K., Munoz-Unceta, P., Kamps, M., Streefland, T., 2019. Process model for the two pilot cases. Construction and demolition waste in the Amsterdam Metropolitan Area. REPAiR Project. Addendum to deliverable D3.3 (unpublished)

Homes and Communities Agencies, 2015. Guidance on dereliction , demolition and remediation costs.

Martinez-Sanchez, V., Kromann, M.A., Astrup, T.F., 2015. Life cycle costing of waste management systems: Overview, calculation principles and case studies. Waste Manag. 36, 343–355. doi:10.1016/j.wasman.2014.10.033.

Raad van State, 2018. Uitspraak 201206132/1/A4 [WWW Document]. URL www.raadvanstate.nl.

Rivas, M., Serafini, J., Glen, J., Angus, A., 2017. Monetising the impacts of waste incinerators sited on brownfield land using the hedonic pricing method. Waste Manag. 61, 608–616. doi:10.1016/j.wasman.2016.10.036.

Taelman, S.E., Sanjuan-delmás, D., Tonini, J., Dewulf, J., Tonini, D., Dewulf, J., 2019. An Operational Framework for Sustainability Assessment Including Local to Global Impacts: Focus on Waste Management Systems. Resour. Conserv. Recycl. 2, 100005. doi:10.1016/j.rcrx.2019.100005.
